# Triboelectric Nanogenerator‐Enabled Digital Twins in Civil Engineering Infrastructure 4.0: A Comprehensive Review

**DOI:** 10.1002/advs.202306574

**Published:** 2024-03-22

**Authors:** Yafeng Pang, Tianyiyi He, Shuainian Liu, Xingyi Zhu, Chengkuo Lee

**Affiliations:** ^1^ Key Laboratory of Road and Traffic Engineering of Ministry of Education Tongji University Shanghai 200092 P. R. China; ^2^ Department of Electrical and Computer Engineering National University of Singapore 4 Engineering Drive 3 Singapore 117576 Singapore; ^3^ Center for Intelligent Sensors and MEMS National University of Singapore Block E6 #05‐11, 5 Engineering Drive 1 Singapore 117608 Singapore; ^4^ National University of Singapore Suzhou Research Institute (NUSRI) Suzhou Industrial Park Suzhou 215123 China

**Keywords:** civil infrastructure, digital twins, energy harvester, self‐powered sensing, triboelectric nanogenerator

## Abstract

The emergence of digital twins has ushered in a new era in civil engineering with a focus on achieving sustainable energy supply, real‐time sensing, and rapid warning systems. These key development goals mean the arrival of Civil Engineering 4.0.The advent of triboelectric nanogenerators (TENGs) demonstrates the feasibility of energy harvesting and self‐powered sensing. This review aims to provide a comprehensive analysis of the fundamental elements comprising civil infrastructure, encompassing various structures such as buildings, pavements, rail tracks, bridges, tunnels, and ports. First, an elaboration is provided on smart engineering structures with digital twins. Following that, the paper examines the impact of using TENG‐enabled strategies on smart civil infrastructure through the integration of materials and structures. The various infrastructures provided by TENGs have been analyzed to identify the key research interest. These areas encompass a wide range of civil infrastructure characteristics, including safety, efficiency, energy conservation, and other related themes. The challenges and future perspectives of TENG‐enabled smart civil infrastructure are briefly discussed in the final section. In conclusion, it is conceivable that in the near future, there will be a proliferation of smart civil infrastructure accompanied by sustainable and comprehensive smart services.

## Graphical abstract

Considering the substantial advantages of TENGs in tackling the energy supply and self‐powered sensing challenges, a guideline for triboelectric nanogenerator enabled digital twins in civil engineering infrastructure is provided, including buildings, pavements, rail tracks, bridges, tunnels, and ports. Finally, the challenges and future perspectives of TENG‐enabled smart civil infrastructure are briefly discussed to drive future development.

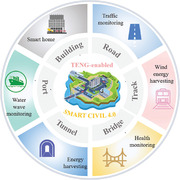

## 1 Introduction

Civil engineering is a general term for the science and technology of constructing various types of engineering infrastructure, covering many fields such as buildings, bridges, roads, tunnels (including undersea tunnels) and port engineering.^[^ ^1^, ^2^ ^]^ With the rapid development of the economy, infrastructure construction has also entered a stage of development from quantitative to qualitative changes. The design, construction, monitoring and operation of a large number of ultra‐high, ultra‐long, ultra‐deep, and ultra‐large projects have put forward higher requirements for civil engineering.^[^ ^3^, ^4^, ^5^ ^]^ A series of problems such as limited data monitoring, high latency, low resource utilization efficiency, insufficient prediction capabilities, difficulty in coping with complexity, sustainability challenges, communication and collaboration limitations, and security limit the capability of any single sensor to meet the demands of modern civil engineering infrastructure. Therefore, the concept of Civil Infrastructure 4.0 came into being to improve efficiency, sustainability and safety.^[^ ^6^, ^7^ ^]^ Civil Infrastructure 4.0 not only includes full life cycle structural health monitoring, but also involves robot‐based automated and intelligent construction, intelligent management of electric energy, water and other resources, sustainability and environmental protection, real‐time decision support, traffic management and user experience and many other key areas.^[^ ^8^, ^9^ ^]^ These requirements are driving the development of civil infrastructure in a smarter, more efficient, and more sustainable direction. At the same time, it also means that the development of smart civil infrastructure 4.0 needs to be driven by the combination of a variety of advanced, intelligent, and digital technologies. No single technology can realize the intelligence and functionality of the entire life cycle of civil infrastructure.

Fortunately, benefiting from the development of sensor edge technology, cloud computing, artificial intelligence (AI),virtual reality (VR), 5G and the Internet of Things(IoTs), the use of digital twin technology to address the challenges of Civil Infrastructure 4.0 is receiving increasing attention.^[^ ^10^, ^11^, ^12^, ^13^, ^14^ ^]^ Digital twins are a concept that integrates real‐world objects or systems with virtual models. The background may be the rapid development of digital technology, the enhancement of computing power, and the rise of big data technology. At the same time, the rise of IoTs enables objects in the physical world to be connected to the internet and transmit data in real time, which provides digital twins with rich real‐time information. In addition, the advances in AI technology, such as machine learning and deep learning, enable digital twins to continuously learn and improve to more accurately simulate the behavior of physical systems.^[^ ^15^, ^16^, ^17^ ^]^ Some essential application scenarios of digital twins include: 1) Civil infrastructure: In civil infrastructure projects, digital twins can be adopted in design, construction and maintenance to improve project quality, safety and efficiency.^[^ ^18^ ^]^ 2) Smart cities: In the construction of smart cities, digital twins can help urban decision‐makers simulate urban infrastructure, optimize urban transportation, energy, and water resources management to improve urban security and sustainable development.^[^ ^19^ ^]^ 3) Energy field: In terms of energy production and distribution, digital twins can optimize the operation of power grids, wind power and solar systems for improving energy efficiency and renewable energy utilization.^[^ ^20^ ^]^ 4) Healthcare: Digital twins can be used to simulate human organs, disease transmission, and drug development, thereby helping doctors formulate better treatment plans and improving the performance and maintenance of medical equipment.^[^ ^21^ ^]^ 5) Military and national defense: Digital twins have important applications in simulating tactical operations, weapon system development and military training, which can reduce risks and improve the effectiveness of military decision‐making.^[^ ^22^ ^]^ 6) Aerospace: The aerospace field can use digital twins to design and test aircraft, optimize flight operations, and improve aviation safety.^[^ ^23^ ^]^ In short, the comprehensive application of the digital twin concept covers various fields and provides great potential for improving efficiency, reducing costs, and improving decision‐making.

However, one vital challenge encountered in the advancement of digital twin technology in civil engineering infrastructure 4.0 pertains to the installation of sensor networks, encompassing sensor units and power sources for energy provision. A multitude of endeavors have been undertaken to harvest energy and facilitate the monitoring of structural integrity to overcome this specific challenge. These endeavors encompass several technologies, including thermoelectric technology,^[^ ^24^, ^25^, ^26^, ^27^ ^]^ magnetic coupling,^[^ ^28^, ^29^, ^30^ ^]^ piezoelectric nanogenerators (PENG),^[^ ^31^, ^32^, ^33^ ^]^ and TENG.^[^ ^34^, ^35^, ^36^, ^37^ ^]^ Due to the instability of thermoelectric technology,^[^ ^38^ ^]^ the size of magnetic coupling^[^ ^39^ ^]^ and the small signal of PENG,^[^ ^40^ ^]^ TENG emerges as a highly auspicious novel technology employed in the realm of digital twins inside the domain of civil engineering infrastructure 4.0. TENG based on the principles of triboelectricity and electrostatic induction was first invented by Prof. Wang in 2012.^[^ ^41^, ^42^ ^]^ Four working modes include the vertical‐contact separation mode,^[^ ^43^, ^44^, ^45^ ^]^ sliding mode,^[^ ^46^, ^47^, ^48^ ^]^ freestanding triboelectric‐layer mode,^[^ ^49^, ^50^, ^51^ ^]^ and single electrode mode.^[^ ^52^, ^53^, ^54^ ^]^ Several power management strategies have been proposed to greatly enhance the output through charge excitation, thereby improving energy harvesting efficiency.^[^ ^55^, ^56^ ^]^ Besides, self‐powered sensing has found considerable application in many domains such as eye tracking, speed, acceleration, pressure, temperature, and humidity monitoring.^[^ ^57^, ^58^, ^59^ ^]^ This popularity can be attributed to its numerous advantages, including a wide range of material options, lightweight design, affordability, a broad operational range, and high efficiency in energy conversion. Energy harvesting is the fundamental operation of a TENG. Its utilization has been extended in several domains, including micro‐nano energy, self‐powered sensing, blue energy, and high‐voltage power supply. These applications have been extensively explored in the literature, as evidenced by numerous studies.^[^ ^60^, ^61^, ^62^, ^63^, ^64^, ^65^, ^66^, ^67^, ^68^, ^69^, ^70^ ^]^ Meantime, it is expected that TENGs will undergo a process of industrialization and find applications in other domains, including IoTs, human‐computer interaction (HMI),^[^ ^71^, ^72^ ^]^ intelligent transportation,^[^ ^73^, ^74^, ^75^ ^]^ civil engineering. This widespread adoption of TENGs has the potential to significantly impact various elements of human social life. TENG is widely acknowledged to have a substantial impact on the implementation of digital twins in the context of civil engineering infrastructure 4.0.

**Figure 1:**
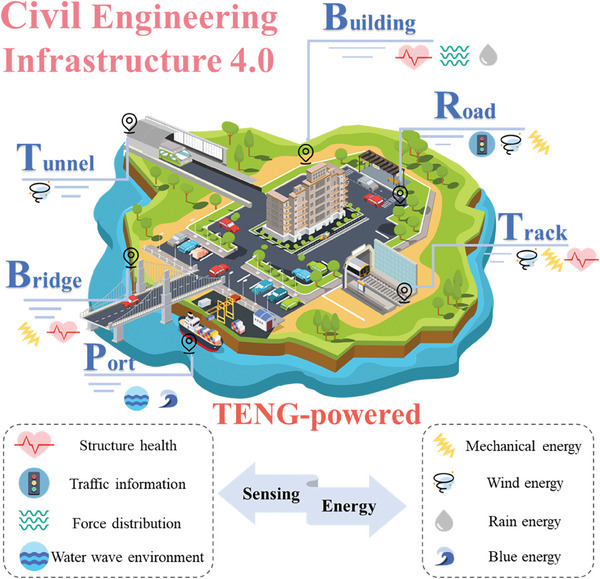
Overview of TENG‐enabled smart civil infrastructure applications relies on self‐powered sensing and energy harvesting technologies.

This study provides a comprehensive overview of the research conducted on TENGs and their diverse applications in civil engineering infrastructure 4.0. The subsequent sections will discuss the research applications of TENGs in civil infrastructure, encompassing smart buildings, smart roads, smart rail tracks, smart bridges, smart tunnels, and smart ports. To gain comprehension of TENG's endeavors in the integrated development of smart civil infrastructure, an initial description of building materials rooted in TENG technology was provided. Subsequently, a comprehensive overview of the many applications of TENG‐enabled self‐powered sensing and energy harvesting technologies in smart civil infrastructure was provided, as seen in **Figure** **1**. In conclusion, this study presents the problems and future possibilities for the implementation of TENG‐enabled digital twins in civil engineering infrastructure 4.0. Hence, the significance of this study lies in its advancement of civil engineering 4.0 by introducing a novel approach that integrates autonomous sensing and energy harvesting capabilities.

## 2 Overview of Smart Civil Engineering and Triboelectric Nanogenerator

### 2.1 Brief Description of Civil Engineering 4.0

Civil engineering 4.0 refers to the comprehensive integration of the “Internet +” idea with the conventional construction engineering domain within the context of a smart city. This integration encompasses multiple aspects, including on‐site production progress of infrastructure and essential equipment and the promotion of environmentally friendly and socially responsible construction practices.^[^ ^76^, ^77^, ^78^ ^]^ A number of new‐generation technologies, such as the Internet of Things, sensing technology, cloud computing, artificial intelligence, and digital twins, were fully implemented to accomplish integrated visual management of the construction site, intelligent interaction, and productive work.^[^ ^79^, ^80^, ^81^, ^82^, ^83^ ^]^ Moreover, the concept of civil infrastructure 4.0 has been put forward as a means to tackle the challenges associated with assessing the durability, safety, reliability, and sustainability of infrastructure. Monitoring and early warning of building structures not only results in significant reductions in maintenance costs but also improves the capacity for prediction.^[^ ^84^, ^85^, ^86^, ^87^ ^]^ In the field of civil engineering, a commonly employed approach for alerting and monitoring the condition of structures is the integration of a network of sensors within the structure itself. This network enables real‐time assessment of the structure's performance.^[^ ^88^, ^89^, ^90^ ^]^ Currently, its primary application is in engineering disciplines such as high‐rise structures, bridges, and dams.^[^ ^91^ ^]^

**Figure 2:**
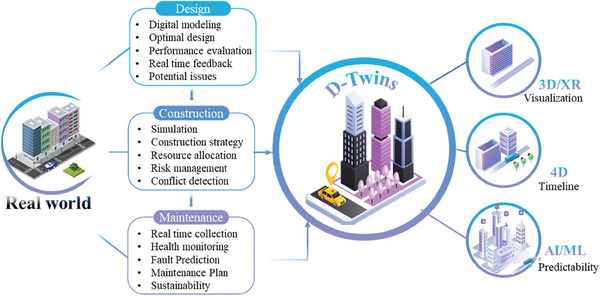
Digital twins in civil engineering 4.0.

The utilization of digital twins in the domain of civil engineering has been progressively growing and gaining widespread acknowledgment. The utilization of digital twin technology is becoming increasingly prevalent in civil engineering projects as a result of advancements in digital technology and enhanced computer capabilities. This technology has proven to be beneficial in enhancing the design, construction, and maintenance processes, as seen in **Figure** **2**.^[^ ^92^, ^93^, ^94^, ^95^, ^96^ ^]^ Digital twins are utilized in the design phase to generate precise digital representations of actual elements. The utilization of past experience data, real‐time data, and simulation techniques can provide valuable support to civil engineers in the optimization of designs, assessment of the performance and behavior of various alternatives, and early identification of potential issues through the use of digital models. Digital twins offer real‐time input throughout the design process, enhancing design decision‐making and guaranteeing the attainment of the most optimal solution. Digital twins have the capability to model and optimize the construction processes of civil engineering projects during the construction phase. By utilizing the capabilities of digital twins, it becomes possible to simulate the impacts of various construction plans and anticipate future disputes and issues beforehand. Hence, the utilization of digital twin technology facilitates the systematic arrangement of construction activities, enhances the efficient allocation of resources, diminishes construction duration and expenses, and enables timely detection and mitigation of potential construction hazards. During the operation and maintenance phases, digital twins play a crucial role in monitoring and assessing the operational condition of civil engineering entities by collecting and analyzing real‐time data. The calibration of real‐time data obtained from the sensory network with the virtual data in the digital twin model is crucial for achieving effective structural health monitoring, accurate equipment failure prediction, and the implementation of suitable maintenance strategies. Furthermore, digital twins help in maintenance planning, the optimization of maintenance strategies, and the improvement of facility reliability and sustainability. Digital twins play an essential role in the design, construction, and operation phases of civil engineering, facilitating full lifecycle support and optimization through real‐time data and model mapping and sharing.^[^ ^97^, ^98^ ^]^

Increasing numbers of embedded or implanted sensors will be incorporated into civil engineering structures in the future to realize the digitization and functionalization of civil engineering infrastructure, making the energy supply of sensors an imperative issue that must be resolved. Hence, the utilization of enhanced sensing technologies for the purpose of effectively harnessing renewable energy within the environment represents the prospective trajectory of civil engineering infrastructure 4.0.

### 2.2 Mechanization and Structure of Triboelectric Nanogenerator

**Figure 3:**
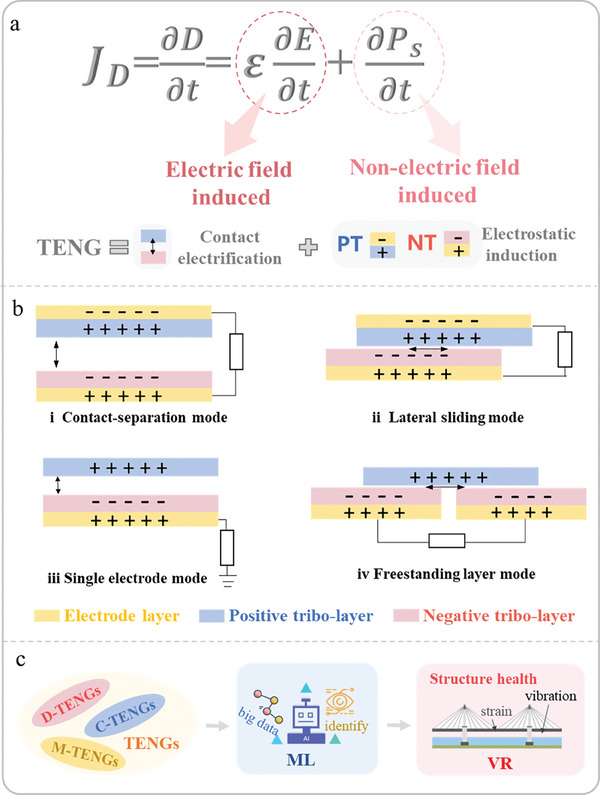
Theory and work mode of triboelectric nanogenrators. a) The working mechanisms of TENGs. b) Four working modes. c) The path to realizing digital twins based on TENGs.

The phenomenon of contact electrification (CE) has a history spanning over 2600 years. However, the mechanism of CE, such as charge transfer and distribution processes, have long posed challenging questions for researchers. Since 2012, the TENG based on the principles of frictional electrification and electrostatic induction was developed, which has garnered significant interest within the domains of energy harvesting and self‐powered sensing.^[^ ^99^ ^]^ The electron transfer caused by overlapping electron clouds dominates the CE. Meanwhile, the electron transfer process of different functional group pairs was investigated in detail to demonstrate the correlation between the molecular structure and macroscopic electrification behavior of polymers.^[^ ^100^ ^]^ The theory of TENG is based on Maxwell's displacement current.^[^ ^101^, ^102^, ^103^ ^]^ In contrast to the generally observed current conveyed by free electrons, displacement current is the current generated by time‐varying (vacuum or medium) electric fields. In the year 2017, Professor Wang made a significant contribution by expanding the concept of displacement current. This expansion involved the introduction of the *P*_s_ term into the potential displacement vector *D*, which subsequently allowed for the derivation of the output power of a nanogenerator.^[^ ^104^ ^]^ The polarization density (*P*_s_) arises from the surface static charge induced by mechanical triggering, and it should be distinguished from the dielectric polarization that results from an electric field. Surface electrostatic charge can be formed through frictional electrification, irrespective of the presence of an external electric field. Furthermore, a novel equation for Maxwell's displacement current has been derived (see **Figure** **3**a).^[^ ^105^, ^106^, ^107^, ^108^ ^]^ This equation consists of two components: the first component represents the displacement current resulting from the time‐varying electric field and material polarization as described by Maxwell, which serves as the theoretical basis for the generation of radio waves. The second component, proposed by Prof. Wang, accounts for the displacement current induced by non‐external electric fields (e.g., charges generated through triboelectric friction or piezoelectric effects), which forms the fundamental principle underlying nanogenerators.

Moreover, the basic principle behind the operation of a TENG can be succinctly described as follows: When the tribo‐layers come into contact with different electron affinities, opposing charges are generated. Subsequently, the presence of opposing charges on the surfaces of two tribo‐layers will give rise to an electric potential as a result of their separation motion. The presence of an external circuit connecting the back electrodes of two tribo‐layer materials results in the flow of electrons across the electrodes. This electron flow is driven by the potential difference, which serves to equalize the electrostatic potential difference across the thin films. Once the two tribo‐friction layers come into contact again, the potential difference generated by the frictional charge dissipates, and electrons flow in reverse.^[^ ^109^, ^110^, ^111^ ^]^ Specifically, the operating mode of TENG consists of contact‐separation mode, lateral sliding mode, single electrode mode, and freestanding layer mode, as shown in Figure 3b, in order to accommodate a variety of applications.

The present summary provides an overview of the implementation of various work modes in smart civil infrastructure. In the contact‐separation mode, the predominant electronic motion takes place either between the tribo‐dielectric layer and the metal electrode or between the dielectric layer and the metal electrode. This method is specifically well‐suited for conducting research on energy harvesting systems implemented on roadways and railway tracks, as well as for the extraction of energy from ocean waves.^[^ ^112^, ^113^ ^]^ For the lateral sliding mode, the mechanical force parallel to the contact surface is required to assure that one tribo‐layer slides over another.^[^ ^114^, ^115^, ^116^ ^]^ Hence, it is notably well‐suited for the extraction of raindrop energy from the rooftops of smart buildings. Furthermore, it is worth noting that the external surface of civil infrastructure remains still, although various modes of transportation, such as automobiles and trains, traverse this external surface. The installation of tribolayers on objects in motion is not a viable option. Hence, it can be inferred that the single electrode mode of TENGs is equally well‐suited for energy harvesting within the primary framework of civil infrastructure.^[^ ^117^, ^118^, ^119^ ^]^ Moreover, in the context of the freestanding layer mode, an autonomous tribo‐layer exhibits unrestricted movement across two stationary tribo‐electrodes that are linked to an external circuit. Electrostatic induction between two fixed electrodes is used to generate power in this mode.^[^ ^120^ ^]^ This mode's power output is determined by the amount of power generated. It is mostly relevant to infrastructure that covers a reasonably large territory, such as speed bumps, roadside infrastructure, smart homes, and other similar applications.

As shown in Figure 3c, TENG‐enabled digital twins can promote the construction of civil infrastructure 4.0, which harvest energy dissipated in the environment, and monitor the structural health condition in real time throughout the entire life cycle of civil infrastructure design, construction, and maintenance, thereby improving safety and efficiency. The following is the path to realize digital twins of civil infrastructure based on TENG. First, a set of integrated TENGs are designed for covering the design (D‐TENGs), construction (C‐TENGs), and maintenance stages (M‐TENGs) of the entire life cycle of civil infrastructure, which can monitor all key information of civil infrastructure in real time. Specifically, D‐TENGs carry out soil and geological condition monitoring, environmental condition monitoring (including meteorological data, temperature, humidity, etc.) and structural load analysis. C‐TENGs monitor the deformation and displacement of infrastructure such as buildings or bridges in real time to ensure safety and quality assurance. Furthermore, material quality control, such as the quality and strength of concrete, steel bars and other materials, is also a focus of C‐TENGs. Besides, M‐TENGs play an irreplaceable role in structural health monitoring, energy and resource utilization, and maintenance demand prediction to extend structural life and reduce unexpected problems. Then, machine learning technology is used to identify, classify and other data mining processes on the large amount of monitored data information to achieve predictions on the environmental meteorology, structural health, operation and maintenance of civil infrastructure. Finally, a demonstration of a digital twin is built to create replicated digital information of the above operations in a virtual reality (VR) environment (i.e., cyberspace). For example, information such as structural deformation, stress distribution and displacement processed by machine learning is used to build a bridge structural health monitoring system based on digital twins to achieve unmanned and automated prediction and management of structural health status.

## 3 TENG‐Enabled Constructions Building Materials

In the field of civil engineering, the activity of structural design is one that is utilized in the process of constructing structures and infrastructure. The implementation of advanced structural design necessitates the adoption of a performance‐based methodology that considers multiple factors such as safety, durability, adaptability, and sustainability of the structure.^[^ ^121^, ^122^, ^123^ ^]^ Consequently, there is an increasing trend toward the adoption of material‐ and performance‐based approaches in structural integration design. The conventional civil infrastructure comprises a composite blend of cement, asphalt, gravel, sand, and other constituent materials in certain proportions. In order to enhance the strength and durability of civil infrastructure, there has been a steady adoption of reinforcing materials, such as basalt fibers and polyethylene fibers, as modifiers.^[^ ^124^, ^125^, ^126^ ^]^ Hence, it is plausible to explore the feasibility of incorporating advanced materials possessing distinct properties to attain the cognitive capabilities of civil infrastructure, such as magnetism and conductivity, into the construction process, thereby enabling the development of structures endowed with genuine self‐awareness. In recent times, there have been endeavors to incorporate TENG‐based materials into civil engineering applications, such as buildings and pavements. These efforts aim to enable energy harvesting and enhance the materials' capacity for information perception.

**Figure 4:**
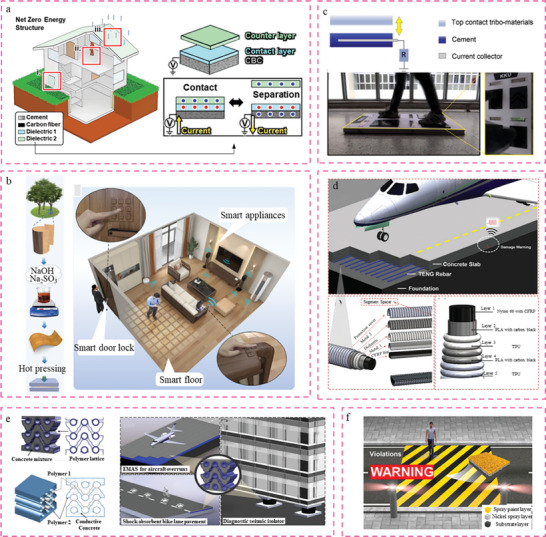
Demonstration of Constructions building materials based TENGs. a) TENG based NZES.Reproduced with permission.^[^ ^130^ ^]^ Copyright 2021,Elsevier. b) Cement‐based TENG with TiO2 nanoparticles.Reproduced with permission.^[^ ^131^ ^]^ Copyright 2022, American Chemical Society. c) Cement‐based TENG with TiO_2_ nanoparticles. Reproduced with permission.^[^ ^135^ ^]^ Copyright 2020, Elsevier. d) TENG‐enabled structural elements for structural health monitoring.Reproduced with permission.^[^ ^140^ ^]^ Copyright 2021,Wiley‐VCH.e) TENG‐based mechanical metamaterial concrete.Reproduced with permission.^[^ ^141^ ^]^ Copyright 2023, Wiley‐VCH. f) Paint based TENG (PBT).Reproduced with permission.^[^ ^144^ ^]^ Copyright 2021,Elsevier.

The concept of net zero energy structures (NZES) has gained significant popularity as a promising alternative for enhancing efficiency and mitigating energy consumption within the building sector across numerous countries.^[^ ^127^, ^128^, ^129^ ^]^ **Figure** **4**a illustrates the use of a TENG‐inspired NZES to harvest renewable energy and reduce energy consumption. This particular system is referred to as cement‐based conductive composite TENGs (CBC‐TENGs) with the inclusion of carbon fiber (CF) fillers.^[^ ^130^ ^]^ CBC serves as an electrode in this NZES system, while FEP is a fixed tribolayer due to its superior electron affinity. In addition to its role in providing structural support, the CBC also serves as a means of energy harvesting. When a residential structure is built using CBC as opposed to traditional cement, it has the potential to harness multiple forms of renewable energy, including wind, biomechanical, and blue energy. Furthermore, the use of CBC in the context of NZES enables the storage of energy inside the structural components. This characteristic expands the potential applications of CBC, positioning it as a comprehensive energy material capable of facilitating the realization of intelligent NZES. Within the realm of smart buildings, smart houses prioritize the well‐being and satisfaction of their occupants to a greater extent. A natural wood‐based triboelectric self‐powered sensor (WTSS) for smart home system are reported in Figure 4b.^[^ ^131^ ^]^ In this study, the wood underwent a chemical treatment method that involved immersion in a solution of NaOH/Na_2_SO_3_, followed by hot pressing. This treatment resulted in the conversion of the wood into a flexible material and the creation of a 3D porous structure with crumpled cell lumina. Furthermore, a Wireless Transceiver Sensor System (WTSS) is widely used in the field of smart homes, with applications that include a self‐powered intelligent password gate control system, smart door locks, and smart floor monitoring. This study highlights the potential uses of the environmentally conscious Wireless Temperature Sensor System (WTSS) in smart buildings, presenting a significant opportunity for the advancement of sustainability in future societies.

Moreover, cement is widely utilized in the field of construction engineering, namely for construction of buildings, bridges, and pavements. This preference is attributed to its widespread availability, notable mechanical strength, affordability, ability to be scaled up, and durability.^[^ ^132^, ^133^, ^134^ ^]^ In the year 2020, a cement‐based TENG with dimensions of 4 × 4 × 1 cm^3^ was designed to capture and convert mechanical energy into usable electrical power, as depicted in Figure 4c.^[^ ^135^ ^]^ The cement‐TiO_2_ nanocomposite incorporates TiO_2_ nanoparticles as a filler material at a weight percentage of 0.2% in Portland cement, leading to a three‐fold enhancement in electrical output in comparison to the original cement TENG. Moreover, the incorporation of TiO_2_ nanoparticles in cement results in a mechanical strength that is 1.3 times higher than that of conventional Portland cement. Furthermore, this modified cement exhibits an energy‐collection capability of 265 mW m^−2^.

The results on dielectric and mechanical properties indicate that adding a small amount of TiO_2_ nanoparticles can significantly improve the energy conversion efficiency and mechanical properties of cement‐TiO2 nanocomposites. Additionally, the cement‐based TENG was enhanced through the integration of carbon black (CB) nanoparticles together with a hydroxyethyl cellulose (HEC) admixture.^[^ ^136^ ^]^ The maximum power density is 2.38 W m^−2^, which is 8.98 times greater than Cement‐TiO_2_‐based TENG. This phenomenon is primarily attributable to the well‐dispersed CB nanoparticles in cement, which increase the dielectric constant via the mechanism of interface polarization. Furthermore, the enhancement of the dielectric constant of composites is facilitated by the decrease in micropores or air voids present in its microstructure. Hence, it can be inferred that the addition of microparticles to cement matrix and enhancing the chemical reaction can lead to an improvement in the output performance of TENG.

Monitoring structural health of civil engineering structures is a formidable task involving structural dynamics, information technology (such as signal transmission, processing, storage, and management), sensor technology, and optimization design.^[^ ^137^, ^138^, ^139^ ^]^ A comprehensive intelligent health monitoring system comprises three essential components: signal acquisition, signal processing, and damage diagnosis. Signal capture is a fundamental and significant aspect of health monitoring, encompassing various essential components. A TENG‐enabled structural elements (TENG‐SEs) with a vertical contact‐separation mode for structure health monitoring (SHM) is illustrated in Figure 4d.^[^ ^140^ ^]^ The TENG‐SE can be described as an intelligent fiber‐reinforced polymer (FPR) rebar that possesses autonomous sensing and reinforcement functionalities. It is composed of many layers, including a central layer, an outer layer, and three internal layers (namely, metal 1, dielectric, and metal 2). The working principle of smart rebar depends mainly on the difference in elongation between cloth and core when subjected to tensile loading. The sensor system generates a potential difference by driving electronics due to the low electric potential of clothes. Tensile tests were done at strain levels of 1% and 2% to assess the mechanical performance and effectiveness of the suggested method for damage detection. It was discovered that the progression of damage can be continuously monitored by observing the changes in TENG‐SE voltage amplitude. In prospective scenarios, the integration of TENG‐SE within civil infrastructure systems holds the potential to establish a versatile, self‐sustaining sensing system. This system can encompass various functionalities, such as: 1) The implementation of intelligent energy harvesting and self‐powered sensing within infrastructure; and 2) Facilitating vehicle‐to‐infrastructure (V2I) communication, particularly in the context of autonomous vehicles (AVs).

Furthermore, a novel nanogenerator mechanical metamaterial concrete was suggested, as depicted in Figure 4e. This concrete consists of auxetic polymer lattices with snap‐through buckling behavior that are completely integrated into a conductive cement matrix.^[^ ^141^ ^]^ The triboelectrification in nanogenerator‐integrated metamaterial concrete can be attributed to the presence of conductive concrete and two polymeric layers. To optimize the construction of prototypes, varying proportions of graphite, ranging from 1% to 4%, were mixed into portland cement. The selection of these percentages was based on considerations of cement workability, mixability, and conductivity. Furthermore, mechanical and metamaterial performance were assessed through the implementation of static and low‐cycle fatigue tests. The findings indicate that nanogenerator's mechanical metamaterial concrete possesses the capability to achieve self‐powered strain and applied load sensing. Furthermore, it is noteworthy that the self‐powered sensing functionality and behaviors of this material remain intact even in the presence of minimal damage. The investigation also encompassed the evaluation of the damage detection capability of metamaterial concrete prototype in the context of SHM applications. This demonstration serves to indicate the significant self‐powered sensing capabilities of the prototype in detecting structural damage, such as fractures and other similar issues. It is important to note that the capabilities of the suggested nanogenerator's mechanical metamaterial concrete extend beyond the aforementioned description. The present study provides an opportunity to explore the potential of sustainable concrete applications in the fields of energy harvesting and self‐powered sensing within the domain of civil engineering. The unique concept offers numerous benefits, such as enhanced strength without adding significant weight, the flexibility to adjust mechanical properties, efficient absorption of energy, adaptability to different sizes, and the capacity to be molded into various shapes. The increasing interest of researchers and engineers is being drawn toward concrete constructions incorporating instrumental metamaterials and infrastructure information self‐awareness. Consequently, the exploration of functional civil infrastructure in the upcoming generation is anticipated to emerge as a prominent area of study.

The novel structure of painted pavement is suitable for intelligent traffic guidance.^[^ ^142^, ^143^ ^]^ A paint‐based triboelectric nanogenerator (PBT) has been created to harness the significant yet underutilized mechanical energy present in pavement infrastructure. This innovative technology utilizes a simple spray deposition technique, incorporating a dielectric layer formed of spray paint, an electrode layer consisting of conductive spray paint, and a substrate (see Figure 4f).^[^ ^144^ ^]^ The PBT was manufactured using a cement substrate to represent the real road and commercially available nickel conductive spray (Super Shield Nickel Conductive Coating, 340 g Aerosol Can, MG Chemicals), graphite spray (Graphite 33 electrically conductive coating, KONTAKT CHEMIE), and spray paint. The deposition of each layer onto the substrate was achieved using a straightforward spray coating technique. Due to the high practicability of the proposed PBT, it has the potential to be implemented in the traffic system of the next generation as well as the security system in the not‐too‐distant future.

Hence, considering the aspects of materials and integrated construction, it is imperative to draw insights from the benefits of TENG in energy harvesting and self‐powered sensing. By utilizing conventional building materials as the foundation and incorporating a limited quantity of functional materials, the construction of net‐zero energy structures or self‐aware structures emerges as an inevitable trend in the development of intelligent civil infrastructure.

## 4 TENG‐Enabled Smart Buildings

The emergence of smart buildings might be seen as an inevitable outcome of the implementation of digital twin technology. The integration of computer technology, communication technology, and building technology enables the realization of an integrated structure, system, service, and function, leading to enhanced efficiency, performance, and productivity.^[^ ^145^, ^146^, ^147^ ^]^ The advancement of smart buildings in civil engineering infrastructure 4.0 necessitates the implementation of effective health monitoring systems, reliable hazards prediction mechanisms, and efficient energy harvesting capabilities. Consequently, an increasing number of sensors are being deployed in smart buildings to accomplish a range of purposes, including prediction, early warning, planning, and guidance. This integration of sensors serves to enhance the safety of building structures, optimize energy utilization, and improve the overall comfort of living environments.^[^ ^148^, ^149^ ^]^ Indeed, there has been a growing interest in the field of smart buildings utilizing triboelectric nanogenerator technology. These buildings present a range of applications, including structural health monitoring, hazard warning systems, energy harvesting capabilities, and smart home functionalities, among others.

**Figure 5:**
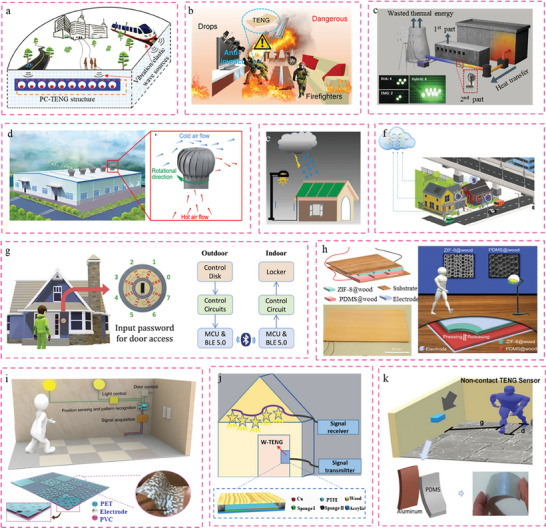
Demonstration of triboelectric nanogenerator enabled smart building. a) A 2D phononic crystal TENG for green buildings. Reproduced with permission.^[^ ^153^ ^]^ Copyright 2022, Elsevier. b) Flame‐retardant triboelectric generator for fire Bluetooth alarm system.Reproduced with permission.^[^ ^157^ ^]^ Copyright 2022, Elsevier.c) Hybridized energy harvesting system based on Stirling engine (HESS).Reproduced with permission.^[^ ^161^ ^]^ Copyright 2021, Elsevier.d) Turbine vent TENG (TV‐TENG) for harvesting irregular wind.Reproduced with permission.^[^ ^168^ ^]^ Copyright 2021, American Chemical Society. e) Liquid‐solid TENG (LS‐TENG) to harvest various forms of water energy.Reproduced with permission.^[^ ^171^ ^]^ Copyright 2022, Elsevier.f) Sound‐driven TENG (SDTENG) for sound monitoring in city.Reproduced with permission.^[^ ^178^ ^]^ Copyright 2022, Wiley‐VCH. g) TENG based control disk interface for human‐ machine interactions in IoT applications.Reproduced with permission.^[^ ^179^ ^]^ Copyright 2020, Elsevier.h) Functionalized wood TENG (FW‐TENG) in smart buildings.Reproduced with permission.^[^ ^180^ ^]^ Copyright 2021, Elsevier. i) Smart floor monitoring system based on TENG mats.Reproduced with permission.^[^ ^176^ ^]^ Copyright 2020, Springer Nature. j) Natural biodegradable wood‐based W‐TENG in smart home.Reproduced with permission.^[^ ^183^ ^]^ Copyright 2020,Elsevier. k) Flexible Non‐Contact Triboelectric Sensors (NCTS) for human subjects moving monitoring.Reproduced with permission.^[^ ^184^ ^]^ Copyright 2021, Elsevier.

SHM in smart buildings is of significant importance as it serves to enhance building safety, mitigate maintenance expenses, and improve sustainability. The distribution of forces within a building is indicative of its overall structural integrity. By implementing real‐time force distribution monitoring and timely warning of anomalous situations, there is potential to significantly mitigate risks to building structures and employees, enhancing overall safety.^[^ ^150^, ^151^, ^152^ ^]^ As depicted in **Figure** **5**a, a 2D phononic crystal TENG (PC‐TENG) system was reported.^[^ ^153^ ^]^ It consists of a periodical spherical cavity type of plate‐like structure and elastic spheres positioned in each spherical cavity. The investigation and optimization of the elastic ball's diameter, material property, surface roughness, and frequency and amplitude of disturbance were specifically focused on using the Hertzian contact model. In the meantime, the PC‐TENG was implanted in the underground foundations of buildings to monitor force disturbances; it had a simple structure, was simple to manufacture, and had a high output performance. Nevertheless, it is imperative to consider some concerns, such as the effect of surface roughness on sensing abilities of PC‐TENG sensor as well as the long‐term durability of this sensor. Hence, it can be posited that TENG holds significant potential as a technology for implementing force distribution in buildings. Moreover, building fires can cause irreparable harm to human life, property safety, and structure of buildings. The occurrence and propagation of building fires are primarily attributable to the extensive use of combustible materials within the structure.^[^ ^154^, ^155^, ^156^ ^]^ Therefore, the suppression and prevention of combustibility have emerged as matters of utmost priority. As depicted in Figure 5b, an anti‐impact and flame retardant elastomers (AFEs)‐based TENG with energy harvesting, safeguarding, and fire alarm performance was manufactured by combining 1 mm 1AFE‐25 (1 AFE contained 25% urea) with 3 mm 6AFE‐25 (6 AFE contained 25% urea).^[^ ^157^ ^]^ The TENG device exhibited consistent mechanical‐electrical‐thermal coupling performance across a range of harsh environments, including anti‐impact and self‐healing capabilities. The potential for enhanced fire danger prevention in future smart buildings is anticipated based on the successful implementation of Bluetooth‐powered alarm systems in portable electronics.

Furthermore, building structure contains a substantial quantity of stored energy. The building energy conservation technology serves as a significant indicator of a nation's progress, while energy recycling plays a crucial role in attaining sustainable development in the realm of buildings.^[^ ^158^, ^159^, ^160^ ^]^ Thermal energy recovery system is a crucial component of modern energy‐efficient structures. The recovery of waste cooling capacity and thermal energy, regardless of whether it is in the form of air, cooling water, or refrigerant, can significantly enhance the energy efficiency of buildings. A hybridized energy harvesting system based on the Stirling engine (HESS), which consists of a disk triboelectric nanogenerator (disk TENG) and an electromagnetic generator (EMG) based on the stirling engine (Figure 5c), is presented with the aim of recovering the wasted thermal energy in industrial applications.^[^ ^161^ ^]^ The disk TENG comprises a rotator and a stator, operating in a freestanding mode to effectively capture and utilize dissipated thermal energy. Moreover, the process of energy harvesting can be effectively transformed into mechanical energy, which can subsequently be converted into electrical energy through the utilization of the LDT Stirling engine. It should be noted that the electrical energy produced by the hybridized nanogenerator was 14,51 times greater than that produced by a single EMG. The findings demonstrate the feasibility of HESS as a potential energy harvesting solution for future industrial heat recovery systems. Wind energy production has been rising substantially in recent years. Similar to solar power generation, wind energy is considered a significant renewable energy source.^[^ ^162^, ^163^ ^]^ Wind energy harvesting, conversion, and reuse have also become popular topics in energy industry. Numerous investigations have been conducted in the field of smart building to explore a range of tactics pertaining to the efficient extraction of wind energy and the measurement of wind speed via TENGs.^[^ ^164^, ^165^, ^166^, ^167^ ^]^ For instance, a turbine vent triboelectric nanogenerator (TV‐TENG) was reported to harvest irregular wind and function as a self‐powered environmental sensing system on the rooftops of buildings.^[^ ^168^ ^]^ The TV‐TENG consists of two freestanding mode TENGs (F‐TENGs) and two contact‐sliding‐separation mode TENGs (CSS‐TENGs), which are placed onto a turbine vent with a volume of ≈0.0064 m^3^. The maximum power output can attain a value of 2.71 mW. Furthermore, an autonomous on‐site industrial monitoring system was designed to monitor temperature and provide safety warnings in industrial settings, as depicted in Figure 5d. This system enhances both environmental sustainability and safety measures within the industrial sector. Furthermore, taking into account the substantial energy derived from natural environment, specifically rainy days,^[^ ^169^, ^170^ ^]^ a liquid‐solid triboelectric nanogenerator (LS‐TENG) was developed to harness diverse forms of water energy. This LS‐TENG exhibits a rapid response upon contact with surrounding water motions (see Figure 5e).^[^ ^171^ ^]^ Several situations were examined including the presence of water droplets, water flow, and the contact of flowing water with the LS‐TENG to showcase its effectiveness in efficiently harnessing water or raindrop energy. Finally, the LS‐TENG can be installed on a huge roof to gather rain droplet energy and power light to investigate the solid‐liquid contact interfaces, which open up the new possibilities for smart home technology.

A smart living environment necessitates smart buildings that implement smart home technology. Data can be collected and analyzed to create a smart scene space with functions such as perception, transmission, memory, reasoning, judgment, and decision‐making using smart building structures that connect furniture and facilities using AIOT technology. This provides users with a humanized living and working environment that is secure, comfortable, healthy, environmentally friendly, and energy efficient. Currently, TENG‐enabled smart homes have been designed to create a secure and comfortable environment.^[^ ^172^, ^173^, ^174^ ^]^ One of the most favored sensing technologies among researchers is TENG, which is empowered by deep learning.^[^ ^175^, ^176^, ^177^ ^]^ The authors proposed an autonomous self‐powered sensor, as depicted in Figure 5f, that utilizes a sound‐driven TENG (SDTENG). This sensor has been successfully combined with deep learning technology to create an intelligent system for sound monitoring and recognition with accuracy of ≈99%.^[^ ^178^ ^]^ In particular, the SDTENG monitors sound levels in a vertical contact‐separation mode, including vehicle noise, children playing, dogs barking, emergency sirens, and street music. This research demonstrates the promising potential of TENGs in the application of USNs.

Furthermore, a control disk interface utilizing a sliding operation, TENG, was created for IoT smart home and access control applications. This interface operates based on a binary coding mechanism and generates a 3‐bit binary‐reflected gray code (BRGC) as shown in Figure 5g.^[^ ^179^ ^]^ Two sensing electrodes were meticulously crafted, one representing the binary digit “0” and the other representing the binary digit “1”. Furthermore, an additional electrode was introduced at the midpoint of the control disk in order to acquire the sliding directions. The utilization of the TENG‐based control disk interface has been implemented in the control of smart home appliances and door access authentication. This application showcases the potential of TENG‐based control interface as a novel means of facilitating human‐machine interactions (HMI) in Internet of Things (IoT). Figure 5h presents a proposed modified wood‐based TENG floor.^[^ ^180^ ^]^ The tribo‐layer materials consist of modified in situ‐grown zeolitic imidazolate framework‐8 (ZIF‐8) and poly(dimethylsiloxane) (PDMS). The electrical signal generation of ZIF‐8 is 80 times greater than that of ordinary native wood. This study demonstrates the enormous potential of functionalized wood TENG (FW‐TENG) in the application of lighting fixtures, calculators, and electrochromic windows in smart structures. Further, a smart floor monitoring system^[^ ^176^ ^]^ comprised of self‐powered triboelectric floor mats and deep learning‐based data analytics was reported, and a unique design with different electrode coverage rates (0%‐100%) was proposed to achieve a balance between clear recognition and the number of floor mats, as shown in Figure 5i. Similarly, an all‐TENG‐based information mat (InfoMat)^[^ ^181^ ^]^ with an in‐home mat array and an entry mat were designed for a digital‐twin smart house. The design of interdigital electrodes enables environmentally insensitive ratio readings from the mat array, thereby eradicating environmental fluctuations. Moreover, the interval arrangement of the mat's pixels permits arbitrary position sensing. It is noteworthy to mention that the array of TENG‐based mats generated in this study demonstrates a remarkable identification accuracy of almost 99%. A floor monitoring system was constructed, consisting of a reference electrode, two coding electrodes, and a sheet electrode. This system demonstrated robustness and intelligence, achieving a high average accuracy of 91.33%.^[^ ^182^ ^]^ Additionally, a W‐TENG utilizing natural New Zealand Pine and polytetrafluoroethylene (PTFE) as triboelectric layers was successfully constructed. This particular W‐TENG operates in a single‐electrode mode and has a power density of 158.2 mW m^−2^. The W‐TENG can be seen as a smart flooring system that can be mounted on a wooden stage floor in order to monitor and document the movements of dancers, as depicted in Figure 5j. In addition, flexible Non‐Contact Triboelectric Sensors (NCTS)^[^ ^183^ ^]^ based on a non‐contact sensing platform can monitor and differentiate intricate movements of subjects without requiring them to wear wearable devices. As depicted in Figure 5k, NCTS was implemented to monitor the movement of multiple human subjects, including their position, speed, frequency, and direction.^[^ ^184^ ^]^ The device possesses the capability to monitor and detect movements within a range of 1.5 meters. Therefore, the proposed non‐contact monitoring strategy has significant benefits for hospital patient movement monitoring.

In summary, the advancement of TENG has facilitated the progress of smart homes, with a particular emphasis on energy harvesting, mechanical distribution perception, and smart home technologies.^[^ ^185^, ^186^ ^]^ The increasing popularity of smart buildings, facilitated by the integration of TENG, is anticipated. These buildings amalgamate structural attributes, encompassing strength, stability, and durability, with functional aspects, including safety and self‐awareness.

## 5 TENG‐Enabled Smart Roads

The urgent demand for road operators and traffic participants is to effectively utilize intelligent technology and digital technology in the construction of smart roads. This includes using smart cities, smart civil infrastructure, and intelligent transportation systems to enhance management efficiency, service quality, and safety while reducing operation and maintenance costs. This demand arises from the need to address the challenges posed by the promotion of intelligent technology in the transportation sector.^[^ ^187^, ^188^, ^189^, ^190^ ^]^ However, a significant challenge that requires attention is the limited integration of road data with other industries, resulting in the prevalent issue of information fragmentation. Generating data linkage with existing smart city systems might be a challenging task.^[^ ^191^, ^192^ ^]^ Hence, the establishment of smart road infrastructure necessitates the implementation of a collaborative, efficient, and all‐encompassing perception system. This system is crucial for facilitating seamless data exchange and integration, thereby fostering the intelligent advancement of road networks. Currently, there is ongoing research on smart highways equipped with TENGs. Energy harvesters utilizing TENG have been increasingly explored for their potential in powering self‐sustaining sensing devices, including those capable of monitoring vehicle speed, vehicle type, and weigh‐in‐motion. These advancements have shown promise in the context of implementing smart road systems. Therefore, TENG emerges as a very promising technology in the realm of establishing information interconnection for smart roads.

Compared to smart buildings, the road network is dense and has a high volume of traffic. In addition to the utilization of natural wind energy, it is worth noting that high‐speed vehicles have the potential to generate significant amounts of wind energy in the vicinity of roadside facilities as a result of their motion.^[^ ^193^, ^194^, ^195^ ^]^ Wind energy harvesting from moving vehicles presents notable advantages in terms of energy quality, storage capacity, and conversion efficiency when compared to the inherent energy found in nature. A miniaturized windmill, known as the ultra‐compact highly efficient miniaturized windmill (MW‐HNG) was designed, which incorporated a hybridized nanogenerator. This nanogenerator combined TENG, piezoelectric nanogenerator (PENG), and electromagnetic generator (EMG). The windmill was designed as a fully enclosed structure using 3D‐printing techniques with the aim of efficiently harnessing natural wind energy. This research was documented in reference.^[^ ^196^ ^]^ It is noteworthy to mention that a hybrid nanogenerator (MW‐HNG) exhibits superior charging performance when compared to the individual generator units. This study highlights the potential of MW‐HNG design as a possible approach for enhancing energy harvesting efficiency. In the realm of actual implementation, it is possible to design the kind, form, and size of a nanogenerator in a manner that aligns with the environmental standards of road infrastructure. This design approach enables the efficient harnessing of the substantial amount of wasted energy available.

**Figure 6:**
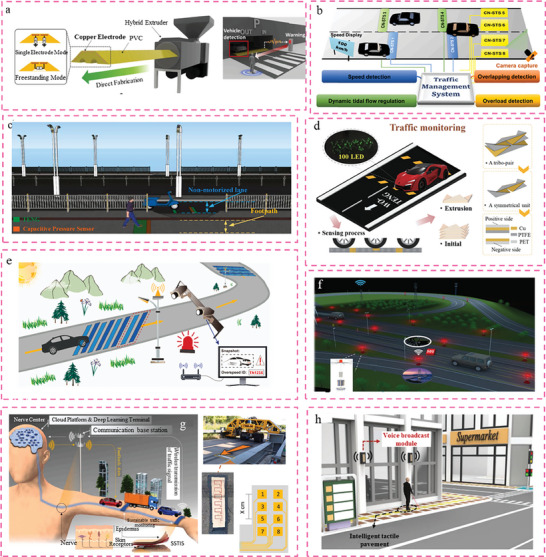
Demonstration of triboelectric nanogenerators enabled smart roads. a) Practical speed bump TENG (SB‐TENG) as a self‐powered automobile warning and velocity sensor. Reproduced with permission.^[^ ^200^ ^]^ Copyright 2020, Elsevier. b) A self‐powered triboelectric sensor (CN‐STS) for smart traffic monitoring and management system.Reproduced with permission.^[^ ^209^ ^]^ Copyright 2022, Elsevier. c) Fully self‐powered TENG (TENG) for wireless traffic monitoring.Reproduced with permission.^[^ ^210^ ^]^ Copyright 2021, Elsevier.d) Waterbomb‐origami‐inspired TENG (WO‐TENG) for traffic environment monitoring.Reproduced with permission.^[^ ^219^ ^]^ Copyright 2022,Springer Nature. e) A self‐powered overspeed wake‐up alarm system (SOWAS) for traffic monitoring.Reproduced with permission.^[^ ^220^ ^]^ Copyright 2023, Elsevier.f) Multi‐Mode TENG for traffic monitoring and warning.Reproduced with permission.^[^ ^221^ ^]^ Copyright 2022, Wiley‐VCH. g) A self‐powered smart transportation infrastructure skin (SSTIS) toward a sustainable monitoring.Reproduced with permission.^[^ ^222^ ^]^ Copyright 2022, Elsevier. h) A self‐powered intelligent voice navigation tactile pavement (SVP).Reproduced with permission.^[^ ^223^ ^]^ Copyright 2022, Wiley‐VCH.

Speed bumps are a crucial component of road infrastructure that serves to prompt drivers to reduce their speed, enhancing overall driving safety. The disregard for the impractical installation of speed bumps is not only associated with their inability to effectively fulfill their intended purpose but also with their potential to impede the efficiency of road traffic. This is primarily due to the distractions they pose to drivers, their potential for improper usage, and the increased risk of traffic accidents.^[^ ^197^, ^198^, ^199^ ^]^ Hence, a revolutionary, self‐aware speed bump was developed, which can proactively deliver pertinent information to the driver will be important in enhancing driving safety. As described in **Figure** **6**a, a proposal was put forth for a practical speed bump TENG (SB‐TENG) by considering current structures, materials, and fabrication processes employed in commercial speed bumps. The SB‐TENG design consists of a commercial PVC speed bump serving as body substrate, along with six copper electrodes.^[^ ^200^ ^]^ This gadget has the capability to operate in both single‐electrode and freestanding modes. The accuracy of the velocity sensor ranges from 4 to 15 km/h^−1^ and can achieve ≈95.001%. Moreover, the SB‐TENG has the potential to serve as a self‐sustaining pedestrian and vehicle alert system, thereby mitigating the occurrence of road accidents.

The issue of traffic safety has consistently been a significant concern among individuals employed in the road and automotive industries. The development of intelligent civil infrastructure has led to an increased focus on safety concerns, resulting in significant human and property losses.^[^ ^201^, ^202^ ^]^ Hence, the study of traffic safety remains an ongoing endeavor, with increasingly stringent demands for safeguarding both human beings and the natural environment. Indeed, the implementation of traffic safety monitoring facilitated by TENG has yielded many outcomes, such as the monitoring of tire pressure^[^ ^203^, ^204^, ^205^, ^206^, ^207^, ^208^ ^]^ and overspeed monitoring, among others. As depicted in Figure 6b, a self‐powered triboelectric sensor (CN‐STS) made of electrospun composite nanofibers was developed for intelligent traffic monitoring and management.^[^ ^209^ ^]^ In the case of CN‐STS, the transferred charge density served as sensing signal for capturing the subtle variations, making it suitable for dynamic traffic monitoring. The incorporation of carbon nanotubes into PVDF nanofibers resulted in improved electrical performance, which had positive effects on the energy storage capabilities and pressure sensitivity of CN‐STS. Furthermore, the utilization of the Alibaba IoT platform facilitated the prompt analysis of the incoming data generated by the CN‐STS sensing system. This advancement can facilitate the analysis of complex road conditions, including the recording of traffic violations and the identification of potential hazards. As shown in Figure 6c, a novel traffic monitoring system utilizing a TENG‐based, fully self‐powered mechanism was proposed for the purpose of monitoring footpaths and non‐motorized roads.^[^ ^210^ ^]^ Using an LC resonant circuit, the developed traffic monitoring system can convert the energy from people's steps and electric motorcycles' turning over into an oscillating signal containing encoded sensing data. In addition, the measured speeds are accurate to a degree exceeding 94%, indicating high practical applicability potential. In other words, this study utilizes the embedded TENG as an active sensor for the first time to address pedestrian crossing safety and non‐motor vehicle safety, thereby expanding the applicability of TENG in the field of intelligent transportation.

Origami structures are used extensively in disciplines such as material science, aerospace, mechanical manufacturing, electronic communications, and medicine because they are designed with special mechanical properties that provide a certain degree of stiffness without the addition of materials.^[^ ^211^, ^212^, ^213^ ^]^ In addition, if an origami structure is manufactured as a TENG, it can substantially increase contact area of the tribo‐layer material without increasing the area that is covered. It indicates that TENG inspired by origami will exhibit an electrical signal that is superior to that of conventional TENG. Because of this, a number of TENGs based on origami were developed.^[^ ^214^, ^215^, ^216^, ^217^, ^218^ ^]^ The application of the origami tessellation‐based TENG (OT‐TENG)^[^ ^108^ ^]^ to intelligent pavement structures was first reported in 2020. The study demonstrates the viability of applying origami‐based TENG to roads for the first time. Then, the waterbomb‐origami‐inspired TENG (WO‐TENG) was developed as a traffic monitoring system,^[^ ^219^ ^]^ which can enhance electrical signals by folding readily (Figure 6d). Due to its low price, small size, light weight, and deformability, the waterbomb origami structure was chosen as a substrate. In order to demonstrate the viability of WO‐TENG for intelligent traffic environment monitoring, the Volkswagen Lavida was utilized to estimate the response of the vehicle passing the packed‐TENG. The results indicate that the accuracy of the weigh‐in motion was 98%. This work demonstrates the further viability of TENG's applicability in smart roads, as well as the benefits of origami structure for enhancing TENG's power generation performance.

As shown in Figure 6e, a self‐powered overspeed wake‐up alarm system (SOWAS) was reported based on TENGs, which are composed of an energy harvesting TENG (E‐TENG), an energy management module (EMM), a fully self‐powered overspeed sensing TENG (S‐TENG), a power switch module (PSM), and a wireless transceiver module (WTM).^[^ ^220^ ^]^ The contribution of this work is to remind us that TENG‐enabled self‐powered monitoring system that consists of many TENG units can be designed to monitor multiple aspects of the intelligent transportation system simultaneously (including energy harvesting, overspeed, dynamic weigh‐in‐motion, etc.), and the different units can be designed according to the actual perception parameter requirements. Similarly, a multi‐mode triboelectric nanogenerator (M‐TENG) was proposed to address traffic safety issues (as seen in Figure 6f).^[^ ^221^ ^]^ Comprised of roller‐based TENG, turnplate‐based TENG, and vertical separation‐based TENG, it can harvest wind energy and mechanical vibrational energy from moving vehicles on roads or bridges. The M‐TENG has exceptional wind energy conversion capabilities and can respond well to the frequency range of bridge vibration in order to power warning lighting and commercial LED strips. After energizing the circuit of the energy management unit, it can also transmit RF signals. The M‐TENG demonstrates its energy harvesting capability and has immense potential in the field of transportation.

As a result of the efforts of numerous researchers, a new era has begun for intelligent, high‐speed nanogenerators. A self‐powered smart transportation infrastructure skin (SSTIS), as shown in Figure 6g, has been proposed to classify traffic in a smart city.^[^ ^222^ ^]^ The SSTIS includes self‐powered, flexible sensors based on the TENG and an artificial intelligence (AI)‐based intelligent analysis system. The feasibility of the SSTIS was evaluated using full‐scale accelerated pavement tests and a deep learning model, attaining a classification accuracy of up to 89.06% for vehicle axle loads. The SSTIS was evaluated on a road using generative adversarial networks (GAN) to augment data, achieving an overall classification accuracy of 81.06 percent for on‐road vehicle types. On the basis of a cloud platform and the Android framework, a mobile system for monitoring traffic signal information was developed. With the accomplishment of full‐scale accelerated pavement tests, the TENG‐enabled smart roads has taken a significant step forward.

Moreover, a distributed self‐powered intelligent voice navigation tactile pavement (SVP)^[^ ^223^ ^]^ based on TENG and an electromagnetic generator (EMG) for blind navigation was proposed to enhance the safety of blind movement (Figure 6h). Composed of an inertial storage hybrid nanogenerator (ISNG), an RF transmitter module, and a voice broadcast module, the SVP can respond to external force incentives within four seconds. Therefore, this work not only enhances the safety of walking for the blind but also functions as a source of energy to power roadside infrastructure.

The TENG‐enabled smart road is believed to play an indispensable role in intelligent transportation systems, smart civil infrastructure, and even the development of smart cities. In the future, harvesting, storing, and utilizing renewable energy such as mechanical energy, thermal energy, and wind energy generated by moving vehicles on roads will occur incrementally as a result of increased research. The implementation of smart cars on smart roads is expected to enhance safety, reliability, and driving comfort. Furthermore, there is a growing interest in enhancing non‐motorized vehicles safety, public transportation, and pedestrian movements. It is anticipated that the integration of TENGs will contribute to the development of more harmonious, efficient, and secure traffic operations in the future.

## 6 TENG‐Enabled Smart Rail Tracks

IoTs and big data technology currently support smart rail tracks, and a variety of self‐powered sensing devices have been developed.^[^ ^224^, ^225^, ^226^ ^]^ Device health monitoring can be accomplished by constructing a perceptual, big data‐based, integrated platform for smart rail transit. Smart rail systems have the capability of utilizing mechanical energy from moving trains and roadside wind energy. This enables the provision of various services such as dynamic data tracking, fault diagnostics, condition monitoring management, and health monitoring for smart rail track systems.^[^ ^227^, ^228^ ^]^ The early development of TENG‐enabled smart train tracks has been achieved with the promotion and implementation of TENG in intelligent transportation systems.

**Figure 7:**
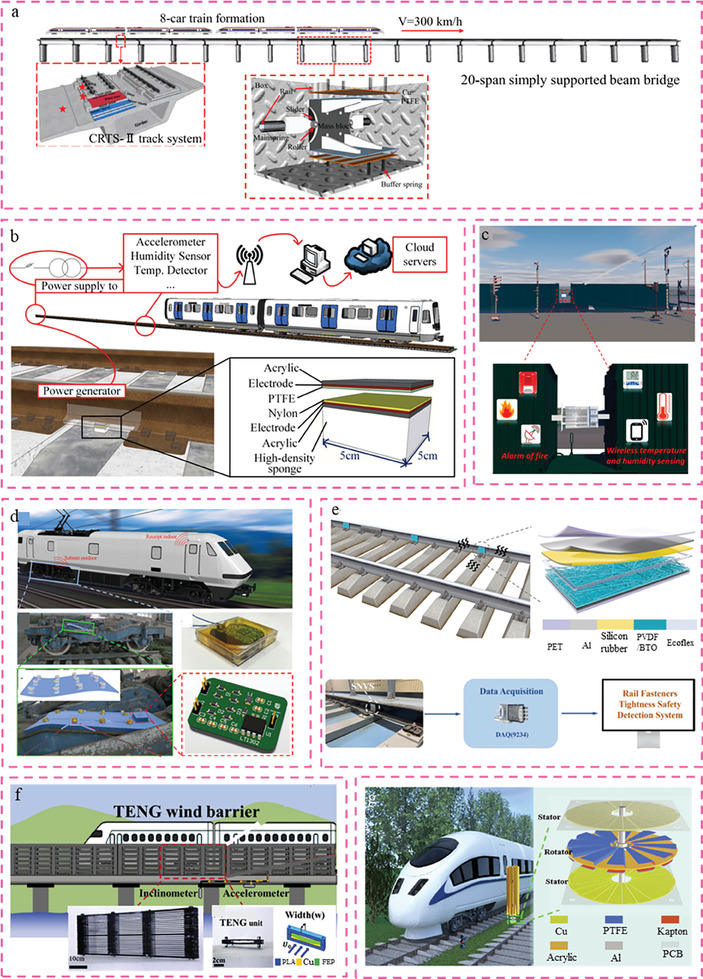
Demonstration of triboelectric nanogenerator enabled smart rail tracks. a) Variable stiffness TENG (VSTENG) for energy harvesting of high‐speed railway.Reproduced with permission.^[^ ^233^ ^]^ Copyright 2022, Elsevier Ltd.b) Sponge‐supported TENG (S‐TENG) for railway health monitoring system.Reproduced with permission.^[^ ^234^ ^]^ Copyright 2021, American Society of Civil Engineers. c) Multiple mode TENG for self‐powered freight train monitoring.Reproduced with permission.^[^ ^235^ ^]^ Copyright 2021, Elsevier.d) Maglev porous nanogenerator (MPNG) or train monitoring system.Reproduced with permission.^[^ ^236^ ^]^ Copyright 2017, Elsevier.e) A self‐powered nanofibers vibration sensor (SNVS) base on TENG for rail fasteners tightness safety detection.Reproduced with permission.^[^ ^237^ ^]^ Copyright 2022, Elsevier.f) Multi‐functional wind barrier integrated by manifold TENG units.Reproduced with permission.^[^ ^242^ ^]^ Copyright 2020, Elsevier.g) Elastic rotation TENG (ER‐TENG) for wind‐energy‐harvesting.Reproduced with permission.^[^ ^243^ ^]^ Copyright 2021, American Chemical Society.

The fast expansion of high‐speed trains has harmed human life and the offspring of wild animals due to vibration and noise pollution.^[^ ^229^, ^230^, ^231^, ^232^ ^]^ The potential consequences of the accumulation of vibration energy within the track structure include the potential for detrimental effects on both the track structure itself and its associated components. The aforementioned factor will significantly impede the efficiency, maintenance, and management of high‐speed railway systems. Therefore, the energy in the track structure is harvested to reduce the damage caused by the accumulation of energy while maximizing refuse utilization. For this reason, as shown in **Figure** **7**a, a variable stiffness TENG (VSTENG)^[^ ^233^ ^]^ was proposed for harvesting vibration energy from high‐speed railways to supply the electrical energy needs of sensor nodes. This VSP is the primary cause of the variable stiffness of VSTENG, and its working principle can be summed up as follows: various vibration excitations will cause the mass block to respond with varying aptness. The aforementioned effect can be achieved through the manipulation of the interval, denoted as “a”, between the two plates of TENGs. Fortunately, the results of the subsequent tests reveal that the VSTENG is able to increase the displacement response and vibrate in a wide frequency domain, thereby creating partial nonlinearity. This variable stiffness design concept merits study and consideration, as it is conducive to improving the coordination and durability of electronic devices and the rail track's primary structure. A sponge‐supported triboelectric nanogenerator (S‐TENG) device was designed to extract mechanical energy from rail vibrations, as depicted in Figure 7b. This device operated in vertical contact mode within the railway health monitoring system.^[^ ^234^ ^]^ The aforementioned study employs a high‐density sponge as a novel and flexible buffer structure under the tribo‐pair. It is noteworthy that the high‐density sponge serves the dual purpose of protecting the tribo‐pair from harm and enhancing the electrical output of the S‐TENG. The significance of this study lies in its ability to establish the viability of utilizing TENGs for the purpose of monitoring the health of railway systems. This is achieved through a comprehensive examination of both theoretical and experimental aspects.

A multimodal triboelectric nanogenerator (MM‐TENG) was developed to address the need for both energy harvesting and operational status monitoring.^[^ ^235^ ^]^ This system consists of multiple sensing units and incorporates a multilayer floating sliding part and a multilayer wave‐shape contact‐separation part. The MM‐TENG was designed to harness the ambient mechanical energy in railway freight transportation, as depicted in Figure 7c. The utilization of energy harvesting can be employed to power the sensors used for monitoring the condition of trains. The multilayer floating sliding component effectively mitigates interface abrasion between tribo‐layers and efficiently captures tiny trigger energies. It is noteworthy that the energy generated by the MM‐TENG is utilized to illuminate a total of 944 light‐emitting diodes (LEDs). Furthermore, it is capable of powering the temperature and humidity meters as well as the fire alarm system, following energy management procedures. This signifies the provision of a reliable power source for the sensor. Similar to the maglev porous nanogenerator (MPNG)^[^ ^236^ ^]^ depicted in Figure 7d, which consists of a TENG and an EMG, it can provide power to a self‐sufficient wireless smart sensor. The integration of the TENG and electromyography (EMG) enables the operational frequency of the motion‐powered nanogenerator (MPNG) to closely align with the frequency of the train during its motion. The research findings suggest that the MPNG has potential applications in wireless monitoring, particularly in the context of train monitoring systems.

In contrast to the aforementioned structural design, an alternative approach can be adopted wherein energy harvesting efficiency or electrical signal performance is enhanced through material modification. A self‐powered nanofiber vibration sensor (SNVS) based on electrospinning nanofibers with high output performance TENG was reported, as depicted in Figure 7e. This sensor has the potential to be manufactured on the rail to enable real‐time detection of rail fastener tightness for safety purposes.^[^ ^237^ ^]^ Septically, the SNVS has a vertical contact‐separation mode. The PVDF/BTO nanofibers containing many nanopores were fabricated using the electrospinning technique, serving as the negatively charged triboelectric layer. During practical implementation, the vibration of the rail induces periodic contact separation motion of SNVS, resulting in the generation of equal amounts of opposing charges on the surfaces of tribo‐materials. Moreover, the electrical output performance remained relatively unaffected even under adverse environmental conditions. This phenomenon demonstrates the viability and longevity of SNVS for track monitoring. Similarly, a novel hybrid self‐sustainable autonomous wireless sensor node (WSN)^[^ ^238^ ^]^ consisting of a PENG and TENG was reported as a self‐sufficient power supply for a track system. The TENG functions practically as an accelerometer sensor, with a good linear sensitivity of 15 V g^−1^ in 0–1.5 g. Moreover, a virtual train was constructed in order to demonstrate the monitoring of aberrant vibrations in rail tracks. The findings indicate the effectiveness of the hybrid self‐sustainable WSN in monitoring the state of trains, including normal, alarm, and overload conditions. Hence, this research suggests the significant potential of hybrid nanogenerators in the field of health monitoring for tracking purposes.

In addition to vibration energy, high‐speed trains' roadside wind energy can produce audible commotion that cannot be ignored. Wind barriers and wind energy harvesting devices is crucial for smart city construction.^[^ ^239^, ^240^, ^241^ ^]^ A novel wind barrier incorporated multiple TENG units was designed.^[^ ^242^ ^]^ This design consists of two copper electrodes and one strip of fluorinated ethylene propylene (FEP) membrane, with their respective ends securely mounted on a 3D printed channel, as depicted in Figure 7f. The wind barrier possesses the capability to not only harness wind energy but also capture slipstream energy generated by passing automobiles. Moreover, the TENG‐based wind barrier is 35% more effective than the traditional porous wind barrier, thereby enhancing the safety of transportation. As depicted in Figure 7g, a wind‐energy‐harvesting device based on an elastic rotation TENG (ER‐TENG)^[^ ^243^ ^]^ was created to harvest the wind energy generated by high‐speed moving trains and power the pertinent signal and sensing devices. In addition, the energy‐harvesting efficiency of the proposed ER‐TENG was increased by a factor of two, while its durability increased by a factor of four when compared to the same characteristics of a conventional rotation sliding triboelectric nanogenerator (RS‐TENG). This was made possible by the rational selection of dielectric materials that had a minimal friction coefficient. Hence, the aforementioned work presents a concept for the large‐scale development of a distributed energy resource.

Significantly, the development of TENG‐enabled smart rail track concentrates on high‐speed railway vibration energy harvesting, wind energy harvesting, slipstream energy from passing vehicles, and the monitoring of railway operation status. It indicates that a significant quantity of renewable energy can be harvested and utilized in the rail network. Moreover, given the pace of trains, monitoring their operational status is also crucial.

## 7 TENG‐Enabled Smart Bridges

The expansion of the global economy has led to an increase in the construction of large‐span bridges. Consequently, it has resulted in a heightened potential risk of vortex‐induced vibration (VIV).^[^ ^244^, ^245^, ^246^ ^]^ The phenomenon of VIV in large‐span bridges has become a significant challenge that requires immediate attention from the engineering community involved in the construction of sea‐crossing bridges. Long‐span bridges exhibit characteristics such as lightweight construction, flexibility, and low structural damping, rendering them susceptible to wind‐induced vibrations. VIV events on suspension bridges have been observed sporadically both domestically and internationally.^[^ ^247^, ^248^, ^249^ ^]^ Hence, the adoption of sophisticated and intelligent strategies becomes imperative in the realm of bridge health monitoring. Vibration changes monitoring in bridge structures enables the prediction and early detection of VIV. This serves the purpose of monitoring the structural integrity of the bridge during VIV events and ensuring the safety of the bridge deck in terms of traffic control.^[^ ^250^, ^251^ ^]^ The power supply of bridge monitoring sensors has emerged as an important challenge to their extensive adoption. The utilization of a passive, self‐powered sensing device for the purpose of monitoring bridge vibration and delivering timely alerts is of utmost importance and requires immediate attention. At present, the implementation of TENG is being extended to the field of bridge health monitoring in order to tackle the challenge of energy provision.

**Figure 8:**
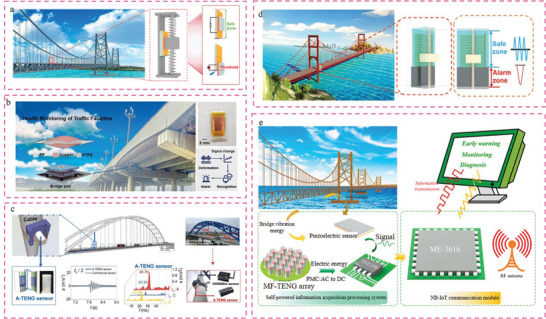
Demonstration of triboelectric nanogenerators enabled smart bridges. a) Dual‐mode TENG (AC/DC‐TENG) as a self‐powered vibration monitoring system.Reproduced with permission.^[^ ^252^ ^]^ Copyright 2020, American Chemical Society.b) Capsule‐shaped TENG (CS‐TENG) for structure health monitoring of bridges.Reproduced with permission.^[^ ^253^ ^]^ Copyright 2022, Americal Chemical Society. c) A self‐driven acceleration TENG sensor (A‐TENG) for acceleration monitoring Reproduced with permission.^[^ ^308^ ^]^ Copyright 2023, Elsevier. d) Alternating‐current TENG (AC‐TENG) for bridge vibration monitoring and safety warning.Reproduced with permission.^[^ ^257^ ^]^ Copyright 2022, Wiley‐VCH.e) Elastic origami TENG (EO‐TENG) as a self‐powered bridge health monitoring system.Reproduced with permission.^[^ ^258^ ^]^ Copyright 2023, Elsevier.

In a recent study, a dual‐mode tribo‐electric nanogenerator (AC/DC‐TENG) was developed to enable the real‐time and continuous detection of vibration characteristics. This nanogenerator, as depicted in **Figure** **8**a, operated by utilizing both alternating current (AC) and direct current (DC) signals. The successful implementation of this technology was reported in a previous publication.^[^ ^252^ ^]^ Continuous monitoring of vibration amplitude and velocity can be achieved by utilizing the electrical signal. Intriguingly, AC and DC modes can be converted into one another; if the amplitude exceeds the danger threshold, AC will be promptly converted to DC, triggering the alarm system to accurately predict construction hazards. Furthermore, a capsule‐shaped TENG (CS‐TENG) was developed to monitor the structural integrity of bridge traffic. This device is depicted in Figure 8b.^[^ ^253^ ^]^ In practice, the CS‐TENG can be mounted on the bridge's support structure to detect deformation and settlement via the electrical signal's varying characteristics. This study showcases the capabilities of TENG in structural health monitoring for bridge facilities.

Strain is a crucial parameter in the context of safety monitoring for bridge structures. Strain monitoring has emerged as a viable approach for facilitating early safety warning and condition evaluation of bridge structures.^[^ ^254^, ^255^ ^]^ A dynamic strain TENG sensor was designed to accurately monitor the time‐dependent behavior of bridges  . This innovative sensor, previously discussed in reference,^[^ ^256^ ^]^ demonstrates its capability to capture the dynamic strain variations experienced by bridges. The accuracy and reliability of the developed TENG are influenced by the frequency of dynamic loading and response amplitude. The research demonstrates that TENG is capable of detecting time‐varying strain responses of a steel bridge in the range of 3 to 150 microstrains with a high precision of 0.1 microstrains. Hence, it is postulated that the utilization of TENG strain sensing technology holds the potential to offer a more extensive and dependable dataset for the purpose of monitoring the structural health of bridges.Besides, a self‐driven acceleration TENG sensor (A‐TENG)^[^ ^308^ ^]^ was reported based on  vibration theory and the TENG mechanism. Comprehensive tests conducted both indoors and on‐site have demonstrated the exceptional performance of the A‐TENG to continuously monitor acceleration with a relative error rate of under 1.5% for natural frequencies(Figure 8c). A novel self‐powered dual‐type signal (DS) sensor was developed by integrating the tribovoltaic nanogenerator (TVNG) with high output and the conventional alternating‐current TENG(AC‐TENG).^[^ ^257^ ^]^ The DS sensor, depicted in Figure 8d, is capable of generating an alternating current (AC) signal within the safe zone. However, once the threshold of the safe zone is exceeded, the sensor produces a direct current (DC) signal and activates a light alarm. The integration of TENGs with traditional vibration nanogenerators (TVNG) serves to underscore the significance of TENG in the realm of self‐powered sensing. The integration of TENG has resulted in enhanced efficiency and expediency in the application of sensors for safety early warning systems. Therefore, this result indicates that the combination of TENG and other sensing mechanisms will be a future trend in self‐powered sensing applications. Furthermore, researchers have successfully developed an elastic origami structure TENG (EO‐TENG) array^[^ ^258^ ^]^ to construct a self‐powered bridge health monitoring system (Figure 8e). This innovative system incorporates an elastic origami structure and is capable of generating a consistent direct current (DC) voltage when connected to an external load. The integration of the self‐powered bridge health data acquisition and processing modular and the NB‐IoT communication modular was implemented in the self‐powered bridge health monitoring system. This system focuses on the monitoring of vibration frequencies and amplitudes using electrical signals obtained from an EO‐TENG. This novel technology holds great potential for advancing the field of bridge health monitoring by enabling self‐powered systems.

## 8 TENG‐Enabled Smart Tunnels

Tunnels possess the capability to reduce distance, safeguard the environment, enhance alignment, and ameliorate road traffic situations. The construction of tunnels has been undertaken in areas characterized by intricate terrain and geological circumstances.^[^ ^259^, ^260^, ^261^ ^]^ Nevertheless, the construction of an underground tunnel presents numerous possible safety dangers that are difficult to forecast. Hence, tunnel engineering is considered a project with a high level of risk. It is necessary to improve the informatization level of tunnel construction to enhance the safety and efficiency of tunnel construction, thereby promoting the development of highway tunnels toward intelligent, integrated, and environmentally sustainable systems.^[^ ^262^, ^263^, ^264^ ^]^ One aspect to consider is that the narrow and elongated configuration of the area facilitates the efficient collection of significant quantities of wind energy, mechanical energy, and other related forms of energy. The practice of harnessing and utilizing energy that would otherwise be squandered has the potential to enhance both the safety and comfort of driving. Additionally, it aligns with the objectives of conserving energy and reducing emissions. Conversely, when peril arises within the tunnel infrastructure, there are significant obstacles in the realm of rescue operations.^[^ ^265^, ^266^ ^]^ Hence, the construction of autonomous perceptual and early warning devices holds considerable implications. TENG is increasingly being utilized in smart tunnels due to its effectiveness in energy harvesting and its ability to power sensing devices.

**Figure 9:**
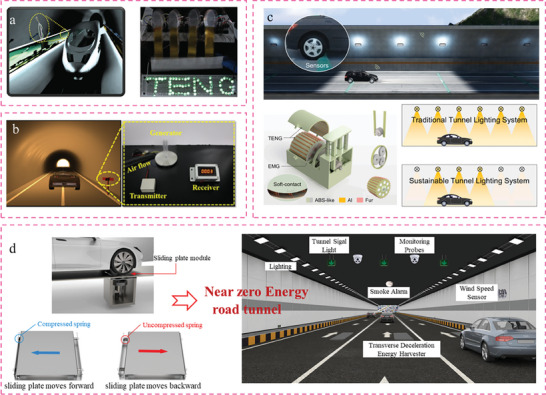
Demonstration of triboelectric nanogenerator enabled smart tunnels. a) Bionic TENG tree for harvesting wind energy in tunnel.Reproduced with permission.^[^ ^269^ ^]^ Copyright 2018, Wiley‐VCH. b) Rotating‐disk‐based hybridized EMG‐TENG for powering wireless traffic volume sensors.Reproduced with permission.^[^ ^273^ ^]^ Copyright 2016, American Chemical Society. c) Mechanical‐regulated and soft‐contact hybridized nanogenerator as sustainable tunnel lighting system.Reproduced with permission.^[^ ^277^ ^]^ Copyright 2022, Elsevier.d) Transverse deceleration energy harvester (TDEH) for road tunnels.Reproduced with permission.^[^ ^281^ ^]^ Copyright 2022, Elsevier.

In tunnels, high‐speed trains have the capacity to create a substantial quantity of wind energy. tunnel under consideration emerges as a highly favorable site for the extraction of wind energy due to its closed structure and extensive mileage.^[^ ^267^, ^268^ ^]^ A bionic TENG tree, as depicted in **Figure** **9**a, which consisted of supercells comprising leaf‐TENG cell I and Stem‐TENG cell II. This innovative design aims to effectively harness wind energy.^[^ ^269^ ^]^ Furthermore, the flexible tree‐structured TENG was implemented in the contact‐separation mode. This TENG was positioned within a lengthy tunnel with the purpose of harnessing wind energy produced by the motion of a subway train and/or a car. By effectively integrating the two cells, the bionic tree‐structure TENG may achieve a peak power output of ≈3.6 mW. The output power is evidently substantial, yet there is uncertainty regarding the energy conversion efficiency between the harvested wind energy and electrical energy. The aforementioned flaw represents an objective that warrants attention in forthcoming applications of TENG within the realm of energy harvesting.

Wireless sensor networks have several notable attributes, including but not limited to low power consumption, affordability, distribution, and self‐organization. These properties render them highly advantageous for monitoring tunnel environments.^[^ ^270^, ^271^, ^272^ ^]^ However, the utilization of wireless sensor networks in tunnel contexts brings attention to their limitations. The development of a rotating‐disk‐based hybridized nanogenerator, which incorporates both a TENG and an EMG, has been undertaken to address the significant difficulties associated with the frequent replacement of batteries in wireless sensors. This nanogenerator is seen in Figure 9b.^[^ ^273^ ^]^ The hybridized nanogenerator functions as an environmentally friendly energy provider for the self‐sustaining active wireless traffic volume sensor. The efficacy of the hybridized nanogenerator in extracting energy from wind created by a mobile vehicle within a tunnel was additionally showcased. This process yielded an output power of 17.5 mW, equating to a power density of 0.557 W m^−2^ when a loading resistance of 700 Ω was applied. The level of power provided is adequate for determining the appropriate functioning of a self‐powered active wireless traffic volume sensor. In subsequent developments, it is imperative to address the relatively low open‐circuit voltage of 3.5 V to enhance the output electrical signal.

The inclusion of tunnel lighting is a crucial aspect of road tunnel infrastructure, as it directly impacts the visibility of the tunnel environment for vehicle drivers. Hence, it is imperative for tunnel illumination design to consider the aspect of driving safety.^[^ ^274^, ^275^, ^276^ ^]^ As depicted in Figure 9c, a mechanically regulated and soft‐contact hybridized nanogenerator (MS‐HNG)^[^ ^277^ ^]^ was developed with the dual functionality of serving as a power source and a distributed traffic monitoring sensor. The MS‐HNG consists of an EMG and a rotatable, free‐standing TENG. The friction force from tribo‐layers was assessed using a force sensor in order to analyze the electrical performance of MS‐HNG in a clever manner. Furthermore, an innovative tunnel lighting system incorporating the feature of “illumination traveling with the vehicle” was successfully implemented in a tunnel. This system demonstrates a significant reduction in energy consumption, ≈28%, when compared to the conventional lighting system, specifically at a traffic flow of 20 km h^−1^. Hence, the aforementioned study facilitates the advancement of conventional tunnel traffic transportation systems through industrial upgrading.

The implementation of road tunnels with near‐zero energy consumption is a crucial approach to attaining energy preservation and mitigating emissions. Efficient harnessing and utilization of renewable energy sources, such as wind energy and mechanical energy from natural environments and moving trains, are essential for the operation of near‐zero‐energy road tunnels. These energy sources are utilized to power the lighting systems in the tunnel, resulting in a significant reduction in energy consumption within the tunnel system.^[^ ^278^, ^279^, ^280^ ^]^ A transverse deceleration energy harvester (TDEH) for near‐zero energy road tunnels is depicted in Figure 9d. The TDEH system consists of a sliding plate, transmission module, and supercapacitor.^[^ ^281^ ^]^ The TDEH has an average mechanical efficiency of ≈45.9%. Furthermore, it is capable of achieving a peak phase power of ≈11 W when subjected to a frequency of 1.5 Hz and an amplitude of 10 mm. The obtained outcome exhibits a notable increase in comparison to energy collector based on TENG technology. It is an undeniable and inexorable trajectory to advance the efficacy of energy harvesting mechanisms and create tunnels with zero energy consumption, utilizing the principles of TENG.

In summary, the research on TENG‐enabled smart tunnels concentrates on high‐speed train vibration energy harvesting, wind energy harvesting, and lighting system power supply. The construction of near‐zero‐energy road tunnels is an inevitable trend, and it is anticipated that TENG will soon make significant contributions to this field.

## 9 TENG‐Enabled Smart Ports

Smart ports encompass the comprehensive utilization of various advanced technologies such as the IoTs, sensor networks, cloud computing, decision analysis optimization, and other smart systems. These technologies enable the efficient execution of tasks such as perception, connectivity, and computation within port management and operations. The ultimate goal is to establish a modern port that is information‐based, intelligent, and optimized.^[^ ^282^, ^283^, ^284^ ^]^ The primary function of smart ports is to autonomously perceive and gather data pertaining to ships, cargo, and port logistics transfer nodes by utilizing IoTs technology. This data serves as the fundamental information for intelligent management and analysis of decisions.^[^ ^285^, ^286^ ^]^ The energy provision for offshore sensors has emerged as an inescapable challenge. Hence, the imperative pursuit of creating a self‐sustaining oceanic sensor capable of catering to port engineering needs and harnessing the potential of oceanic energy for electricity generation emerges as an essential trajectory for the advancement of intelligent ports in the future. The significant utilization of TENG in the domain of blue energy has provided a viable and efficient resolution.^[^ ^287^, ^288^, ^289^, ^290^ ^]^

In a study conducted by Chen et al.,^[^ ^291^ ^]^ a network of TENGs (TENG‐NW) was introduced as a means to efficiently harness kinetic water energy on a wide scale. This network demonstrates the ability to transform various types of motion, including slow, random, and intense oscillatory motion, into electrical energy. In addition, it offers a substantial average power production of 1.15 MW per square kilometer of surface area. This study suggests that there is significant potential for harnessing wave energy through the utilization of contact electrification and electrostatic induction in combination. Furthermore, a hexagonal network of TENGs was devised, drawing inspiration from spherical TENG units.^[^ ^292^ ^]^ This network has seven units positioned at the center of a hexagonal arrangement, with six units situated at the vertices. A self‐powered system utilizing the motion of water waves was built, consisting of a network of water‐wave‐driven TENGs and functional circuits. According to the data provided, the maximum power is ≈12.2 mW (corresponding to a power density of 1.94 W m^−2^).

**Figure 10:**
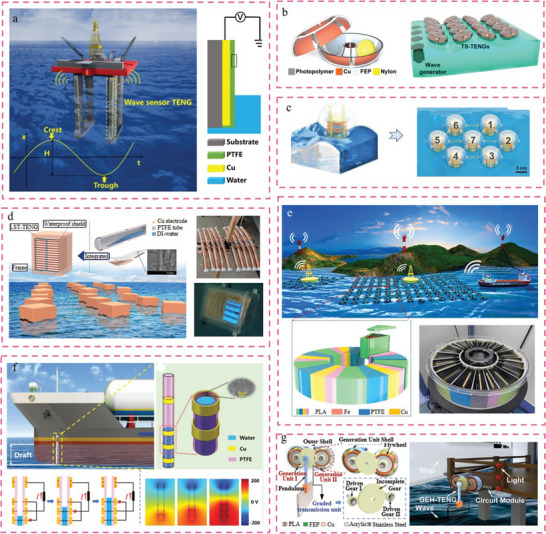
Demonstration of triboelectric nanogenerator enabled smart ports. a) Wave sensor based on liquid‐solid interfacing TENG (WS‐TENG) to monitor wave information.Reproduced with permission.^[^ ^296^ ^]^ Copyright 2019, Elsevier.b) Torus structured TENG (TS‐TENG) for wave energy harvesting.Reproduced with permission.^[^ ^298^ ^]^ Copyright 2019, Elsevier.c )TENG with charge excitation circuit (CEC).Reproduced with permission.^[^ ^299^ ^]^ Copyright 2020, Wiley‐VCH.d) Liquid‐solid tubular TENG (LST‐TENG) arrays for harvesting water wave energy.Reproduced with permission.^[^ ^300^ ^]^ Copyright 2022, Elsevier.e) O‐ring‐like TENG (O‐TENG) for omnidirectional wave energy harvesting.Reproduced with permission.^[^ ^301^ ^]^ Copyright 2022, Elsevier. f) Novel water level sensor based on liquid–solid tubular TENG (LST‐TENG) for ship draft detecting.Reproduced with permission.^[^ ^304^ ^]^ Copyright 2019, Wiley‐VCH.g) Graded energy harvesting TENG (GEH‐TENG), consists of an outer shell, a pendulum, two generation units (I and II) and graded transmission unit.Reproduced with permission.^[^ ^306^ ^]^ Copyright 2021, American Chemical Society.

Furthermore, wave monitoring holds significant importance in various domains such as ocean engineering construction, resource development and use, environmental protection, marine safety, and ocean catastrophe warning.^[^ ^293^, ^294^, ^295^ ^]^ Enhancing the environmental perception capability of smart ports is a crucial strategy for effectively improving the operational, maintenance, and management capacities of port facilities. The development of a wave sensor, known as the liquid‐solid interfacing TENG (WS‐TENG), is illustrated in **Figure** **10**a. This sensor was designed specifically for the purpose of monitoring wave information in the vicinity of naval equipment.^[^ ^296^ ^]^ Based on an investigation into the impact of sensor substrate, wave height, frequency, and salinity on the output performance of the Wave‐Driven TENG (WS‐TENG), it was determined that the WS‐TENG possesses the capability to effectively monitor wave heights at the millimeter scale. One notable observation is that the sensitivity of the WS‐TENG can reach ≈23.5 mV mm^−1^ when the electrode width is set at 10 mm. This phenomenon signifies that the newly developed WS‐TENG has the potential to serve as a viable option for intelligent ocean equipment in the monitoring of waves.

To enhance the efficiency of liquid fuel utilization, the researchers focused on optimizing the wave‐energy‐driven CO2RR system.^[^ ^297^ ^]^ This system consisted of two key components: the capacitance of the triboelectric charge storage device and the operation voltage of the electrochemical cell. The objective was to achieve a near 100% faradaic efficiency (FE). According to the field experiments conducted, it has been observed that the CO2RR system driven by wave energy has the capability to generate a daily yield of 0.325 mmol of formic acid when subjected to a wind speed of 18 knots. This study primarily focuses on the development of a wave‐energy‐driven CO2RR system, offering valuable insights into the optimization of liquid fuel generation. The authors presented a torus‐structured TENG (TS‐TENG) composed of two semi‐torus shells and a rolling nylon ball, as depicted in Figure 10b.^[^ ^298^ ^]^ The conversion of water wave energy into electrical energy is facilitated by the presence of nylon balls enclosed within semi‐torus shells, which are excited by the water waves. In addition, the TS‐TENG units underwent initial rectification on an individual basis before being interconnected in parallel to create an array for the purpose of harnessing water wave energy. The TS‐TENG has a peak power density of 0.21 W m^−2^, characterized by a frequency of 2 Hz and an oscillation angle of 5°. Subsequently, a charge excitation circuit (CEC) was specifically designed to harness water wave energy using a TENG.^[^ ^299^ ^]^ This TENG demonstrated a remarkable power output, reaching a high of 25.8 mW (corresponding to a power density of 3.89 W m^−2^). Furthermore, the current generated by this TENG was significantly amplified, measuring 25.1 mA, which represents a 208‐fold enhancement (see Figure 10c). The field of electrical and computer engineering (ECE) offered valuable insights and direction in the development of an offshore IoT infrastructure.

A high‐output array of LST‐TENG^[^ ^300^ ^]^ was reported based on the contact electrification between PTFE and water, as depicted in Figure 10d. The experimental evaluation involved testing the output performance of the LST‐TENG at various places within the enclosure while subjecting it to varying angles and amplitudes of swing motion. The results indicated that the ideal configuration for the LST‐TENG parallel array yielded peak output power was 215 µW (corresponding to a power density of 68.4 mW m^−2^). This study represents the initial exploration into the distinct sensor movements induced by wave excitation, achieved by integrating LST‐TENG units. This breakthrough marks the commencement of a novel era in on‐site ocean wave energy harvesting.

An O‐ring‐like TENG (O‐TENG) was designed with a modular design, which consists of flexible splicing of fan‐ring‐shaped TENG blocks at various angles.^[^ ^301^ ^]^ This design enables omnidirectional wave energy collection, as depicted in Figure 10e. The O‐TENG possesses the ability to adjust response spectrum in accordance with the prevailing environmental conditions, via its clever design. The O‐TENG has a remarkable capacity to generate a high output capability of 29.90 µC by splicing due to its small internal structure. This is attributed to its exceptional usage of space, which offers novel opportunities for the efficient harvesting of wave energy in several directions. Additionally, a novel monodirectional continuous spinning TENG (CS‐TENG) was introduced to efficiently harness energy from irregular, gentle waves^[^ ^302^ ^]^. Dual symmetry breaking technique was implemented to convert irregular wave excitations into internal unidirectional motions, utilizing non‐mirrored network linkers and a one‐way bearing‐enabled ratchet effect. It is noteworthy that the utilization of an inertia‐wheel‐based energy caching approach is employed to capture the energy from each transient excitation, thereby facilitating an extended duration of rotational motion. In other words, the CS‐TENG, which is artificially produced, has the ability to gather irregular wave excitation to achieve a sustained and intermittent increase in output, thus enhancing its overall performance with peak power of 14.357 mW (corresponding to a power density of 6.9 W m^−2^). Hence, this study presents a significant novel approach toward the development of more efficient energy harvesting systems that include intrinsic adaptability.

In addition to blue energy harvesting, water wave environments monitoring, and risk warning have emerged as prominent areas of research to safeguard the safety and integrity of water environments. Hence, a TENG (SWTENG) was developed with a spherical shape and high symmetry in three dimensions, which can harvest water waves energy and monitor many aspects of the water environment, such as water temperature, water level, and water pollution.^[^ ^303^ ^]^ The aforementioned project has made a significant advancement in the transition of TENGs from the field of blue energy harvesting to the domain of water environment monitoring. Subsequently, a novel self‐sustained water level sensor utilizing a LST‐TENG was developed, exhibiting a remarkable precision of 10 mm, surpassing the conventional draft mark on ships by a factor of ten (refer to Figure 10f).^[^ ^304^ ^]^ Additionally, a novel triboelectric ocean‐wave spectrum sensor (TOSS) was introduced, consisting of a TENG and a hollow ball buoy.^[^ ^305^ ^]^ This sensor offers the capability to monitor water waves on the ocean surface from various directions while mitigating the influence of seawater on its performance. Fortunately, the TOSS exhibits an ultrahigh sensitivity of 2530 mV mm^−1^, which is 100 times greater than that observed in prior studies. Figure 10g demonstrates the fabrication of a graded energy harvesting TENG (GEH‐TENG) for monitoring water wave conditions.^[^ ^306^ ^]^ This device is capable of generating 0.7 mJ of energy during a single operating cycle, which is 2.3 times greater than the energy generated by a non‐graded counterpart. The aforementioned work offers valuable insights into the actual implementation of TENGs for monitoring ocean wave conditions.

In the studies examined, numerous strategies have been implemented to enhance the efficacy of wave energy extraction through the optimization of device structure and array arrangement. These efforts are driven by the abundant resources, substantial reserves, renewable nature, environmental friendliness, and absence of pollution associated with blue energy. In addition, the implementation of TENG‐enabled smart ports has led to a growing emphasis on various monitoring techniques, including wave motion monitoring, water level monitoring, water pollution monitoring, and chemical monitoring. These monitoring practices have become increasingly prominent in the field as they play a crucial role in preventing the occurrence of ocean disasters by ensuring reliable data collection and analysis.

## 10 Challenges and Perspective

Promoting economic and social development and improving people's standards of living depend significantly on the construction and reliable execution of civil infrastructure. The optimal development of civil infrastructure necessitates comprehensive management across its entire lifecycle, encompassing aspects such as materials, structures, and performance. The widespread utilization of TENGs within the engineering discipline has garnered the interest of civil engineers. Is there an optimal approach for developing an integrated smart civil infrastructure? Within this framework, the civil infrastructure possesses the ability to autonomously perceive its own health condition, all the while upholding exceptional mechanical characteristics and effectively gathering and storing energy. This smart civil infrastructure possesses exceptional mechanical performance, durability, and self‐powered sensing capabilities. Hence, this review provides a comprehensive overview of the current utilization of TENGs in the context of smart civil infrastructure. Specifically, it focuses on their application in several domains, including construction building materials, smart buildings, smart roads, smart rail tracks, smart bridges, smart tunnels, and smart ports. The integration of TENG technology has facilitated the incorporation of energy harvesting and self‐powered sensing capabilities into smart civil infrastructure, thereby driving the evolution of a new generation of multifunctional smart civil infrastructure. Furthermore, it is arguable that the implementation of TENG‐enabled smart civil infrastructure would enhance the development of smart cities in the foreseeable future, ushering in a novel era characterized by environmentally friendly, technologically advanced, and secure urban environments.

When considering the collective findings, it becomes evident that there is ample opportunity for more advancements in the development of smart civil infrastructure utilizing TENGs. Despite the notable advancements made in the field of energy harvesting and self‐powered sensing using TENG, there are still significant challenges that must be addressed for practical applications. These challenges include enhancing energy harvesting efficiency, mitigating the effects of temperature and humidity on TENG performance, ensuring the stability of sensing signals, improving durability, and developing effective packaging technology. In real‐world circumstances, addressing the challenges of deformation coordination and structural matching between the TENG integrated inside civil infrastructure and the primary structure is a complex task that requires careful consideration. Hence, the advancement of TENG‐enabled smart civil infrastructure building necessitates collaborative endeavors among academics, indicating that there is still considerable progress to be made in this field.

**Figure 11:**
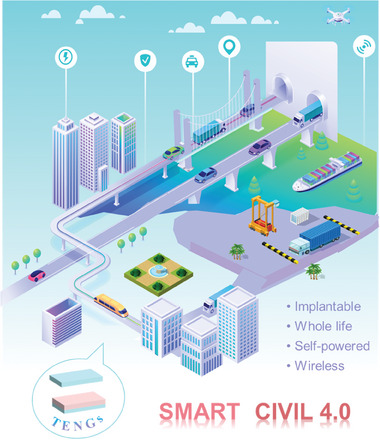
Perspectives of TENG‐enabled smart civil infrastructure 4.0.

The utilization of TENGs in civil engineering infrastructure 4.0 would facilitate the development of digital twins that exhibit enhanced degrees of intelligence, autonomation, and digitization. The integration of sensor networks into the infrastructure will be crucial for enhancing the efficiency, safety, and sustainability of infrastructure operation and management. The design of sensing devices is not solely confined to the materials and structures commonly associated with traditional flexible electronics. Instead, it extends to the civil infrastructure itself, using existing infrastructure substrates as raw materials. By incorporating the principles of flexible electronics, the development of sensing devices tailored for civil engineering applications is made possible. However, a multitude of sensing device networks employ autonomous energy sources, such as solar energy or ambient vibration energy, in order to mitigate dependence on conventional energy sources and enhance the sustainability of sensor systems. Hence, it can be inferred that the forthcoming digital twin civil infrastructure 4.0, facilitated by TNEGs, will incorporate the integration of information perception, analysis, and transmission. This integration will enable the virtual monitoring of the health condition of infrastructure entities in real‐time within a virtual environment. Furthermore, it will allow for the prediction of unforeseen risks based on the spatiotemporal evolution of performance, as depicted in **Figure** **11**. Certainly, numerous challenges persist in achieving the aforementioned multi‐functionality and intelligence, encompassing:

1. *Complexity and reliability*: The integration of self‐powered sensors into civil infrastructure is a multifaceted task. The exceptional functional attributes exhibited by sensors, including precision, dependability, and enduring stability, pose an enormous challenge due to their requirement to function effectively in adverse environmental circumstances. Furthermore, these sensors may encounter challenges related to physical damage, power supply discrepancies, and the preservation of data transmission reliability.
2. *Data processing and analysis*: The processing and analysis of a substantial volume of data produced by self‐powered sensors are required. The process includes the optimization of data storage, real‐time data processing, and the advancement of algorithms that can effectively extract valuable information and provide decision support from diverse data sources.
3. *Standardization and interoperability*: The successful application of self‐powered sensors requires the establishment of appropriate standards and protocols to ensure interoperability and data consistency among different sensors. Standardization plays a crucial role in enabling the seamless integration of diverse devices and systems, thereby promoting consistent data exchange and facilitating collaborative work.
4. *Cost and sustainability*: The manufacture, installation, maintenance, and renewal of sensors require a lot of resources. In order to ensure the sustainability and prolonged functionality of sensors, it is imperative to address key concerns such as cost‐effectiveness, energy supply, and recyclability.

With the promotion and implementation of cutting‐edge technologies, such as TENG and artificial intelligence, in civil engineering, civil engineering is becoming increasingly progressive. There is a prevailing belief that forthcoming civil infrastructure will undergo comprehensive advancements in the domains of energy harvesting and self‐powered sensing. The development of both residential structures and transportation infrastructure will progress toward greater intelligence. In essence, the integration of TENG technology into smart civil infrastructure is poised to have a profound impact on several facets of human existence in the foreseeable future.

It is plausible that the integration of structural health monitoring during the maintenance phase of civil infrastructure will pave the way for the realization of digital twins. Undoubtedly, structural health constitutes a pivotal aspect of civil engineering, and ongoing extensive research in structural performance monitoring and prediction underscores its significance. This progress is marked by the evolution of acquisition devices, the refinement of data acquisition and analysis methods, and the establishment of sophisticated monitoring algorithms and models tailored for structural health. Furthermore, the implementation of digital twins in structural health monitoring follows a relatively uncomplicated technical trajectory, rendering their application more straightforward and feasible. Simultaneously, the paramount importance of ensuring the safety and reliability of infrastructure resonates deeply within society and the economy. Digital twin technology emerges as a proactive tool, allowing timely identification of potential issues, thereby enhancing the efficiency of maintenance and management, and ultimately curbing repair costs and associated risks. Considering these factors collectively, structural health monitoring appears poised to be at the forefront of realizing digital twins, offering advanced and efficient solutions for the operation and maintenance of civil infrastructure.

## Conflict of Interest

The authors declare no conflict of interest.
